# Breast cancer: pathogenesis and treatments

**DOI:** 10.1038/s41392-024-02108-4

**Published:** 2025-02-19

**Authors:** Xin Xiong, Le-Wei Zheng, Yu Ding, Yu-Fei Chen, Yu-Wen Cai, Lei-Ping Wang, Liang Huang, Cui-Cui Liu, Zhi-Ming Shao, Ke-Da Yu

**Affiliations:** 1 Department of Breast Surgery, Key Laboratory of Breast Cancer in Shanghai, Cancer Institute, Fudan University Shanghai Cancer Center, Department of Oncology, Shanghai Medical College, Fudan University, Shanghai, P. R. China; 2 Department of Breast and Thyroid, Guiyang Maternal and Child Health Care Hospital & Guiyang Children’s Hospital, Guiyang, P. R. China; 3 Department of Clinical Medicine, Guizhou Medical University, Guiyang, P. R. China; 4 Department of Breast and Urologic Medical Oncology, Fudan University Shanghai Cancer Center, Department of Oncology, Shanghai Medical College, Fudan University, Shanghai, P. R. China

**Keywords:** Breast cancer, Cancer therapy, Cancer epidemiology, Tumour heterogeneity, Metastasis

## Abstract

Breast cancer, characterized by unique epidemiological patterns and significant heterogeneity, remains one of the leading causes of malignancy-related deaths in women. The increasingly nuanced molecular subtypes of breast cancer have enhanced the comprehension and precision treatment of this disease. The mechanisms of tumorigenesis and progression of breast cancer have been central to scientific research, with investigations spanning various perspectives such as tumor stemness, intra-tumoral microbiota, and circadian rhythms. Technological advancements, particularly those integrated with artificial intelligence, have significantly improved the accuracy of breast cancer detection and diagnosis. The emergence of novel therapeutic concepts and drugs represents a paradigm shift towards personalized medicine. Evidence suggests that optimal diagnosis and treatment models tailored to individual patient risk and expected subtypes are crucial, supporting the era of precision oncology for breast cancer. Despite the rapid advancements in oncology and the increasing emphasis on the clinical precision treatment of breast cancer, a comprehensive update and summary of the panoramic knowledge related to this disease are needed. In this review, we provide a thorough overview of the global status of breast cancer, including its epidemiology, risk factors, pathophysiology, and molecular subtyping. Additionally, we elaborate on the latest research into mechanisms contributing to breast cancer progression, emerging treatment strategies, and long-term patient management. This review offers valuable insights into the latest advancements in Breast Cancer Research, thereby facilitating future progress in both basic research and clinical application.

## Introduction

Breast cancer remains a formidable adversary in the landscape of global health challenges, with its intricate pathogenesis and diverse clinical manifestations posing significant obstacles to effective treatment and prevention.^1–3^ As the global incidence of this disease continues to rise,^4^ it is imperative to unravel the multifaceted nature of breast cancer to develop effective therapeutic strategies.

Despite advancements in early detection and therapeutic strategies, the disease exhibits a complex etiology that necessitates a deeper understanding of its molecular underpinnings and risk factors. There are many factors affecting the tumorigenesis of breast cancer, and evidence illustrates the intricate interplay of genetic, environmental, and lifestyle factors that contribute to that process.^5–7^ Understanding these factors can help in breast cancer prevention and early detection. In addition, the progression of tumor is influenced by various factors operating through distinct mechanisms (such as tumor stemness, intra-tumoral microbiota, and circadian rhythms), and a comprehensive investigation into these mechanisms is essential for identifying potential clinical therapeutic targets.^8^

With the continuous advancements in experimental techniques and sequencing technology, significant progress has been made in the detection and diagnosis of tumor. For example, the combination of liquid biopsy and high-throughput sequencing technology has opened new avenues for cancer diagnosis.^9,10^ Artificial intelligence (AI) is revolutionizing clinical oncology, with considerable potential to improve early tumor detection and risk assessment and to enable more accurate personalized treatment recommendations.^11–14^ The application of these emerging diagnostic methods in breast cancer will be discussed in this review. The traditional treatments for breast cancer include surgery, chemotherapy, radiotherapy, endocrine therapy, targeted therapy, and other related approaches.^15^ In recent years, the advent of precision medicine has set the stage for a new era in breast cancer treatment, with an emphasis on tailored therapies that target the specific molecular characteristics of individual tumors.^16^ Additionally, long-term management of patients with tumors, including breast cancer, is crucial as it directly impacts patients’ quality of life and survival time.^17–19^

**Fig. 1:**
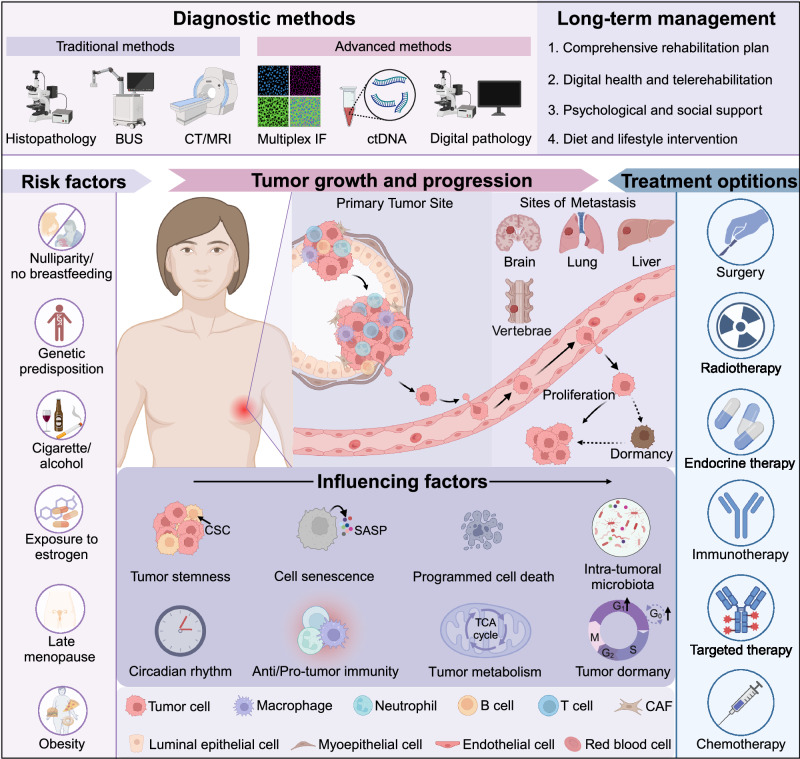
Comprehensive overview of breast cancer pathogenesis and treatment. Breast cancer is one of the most prevalent tumors in women, and its occurrence is associated with a multitude of factors, such as genetic mutations, late menopause, and obesity. The progression of breast cancer is shaped by numerous factors, encompassing both tumor cell characteristics and elements within the tumor microenvironment, whether cellular or non-cellular. In recent years, there have been significant advancements in diagnostic technologies for breast cancer. Alongside traditional imaging techniques and pathological diagnosis methods, liquid biopsy, and multiple immunofluorescence assays, digital pathology approaches are gradually being incorporated into clinical practice. Treatment options for breast cancer are diverse, and recent clinical studies emphasize the importance of individualized and precision treatments. Long-term follow-up management of breast cancer patients is also crucial, as it may impact both the therapeutic outcomes and enhancing patients’ quality of life. BUS B-scan ultrasonography, CT computed tomography, MRI magnetic resonance imaging, IF immunofluorescence, ctDNA circulating tumor DNA, CSC cancer stem cell, SASP senescence-associated secretory phenotype, TCA cycle tricarboxylic acid cycle. The figure was created with Biorender.com

Tracking the latest research advancements is crucial for deepening our understanding of breast cancer and enhancing treatment outcomes for patients. This comprehensive review provides a synthesis of the latest current knowledge, focusing on recent breakthroughs and emerging trends in the pathogenesis, progression, diagnostics, treatment, and follow-up management of breast cancer (Fig. 1).

## Epidemiology and risk factors of breast cancer

Breast cancer is a heterogeneous disease with distinct subtypes characterized by unique epidemiological patterns.^20^ Globally, breast cancer accounts for roughly one-third of all malignancies in women, with its mortality rate constituting about 15% of the total number of cases diagnosed.^4,21^ A complex interplay of genetic, environmental, and lifestyle factors influences the global distribution of breast cancer. High-income countries typically exhibit higher incidence rates than low- and middle-income countries, although mortality rates are often lower due to better access to early detection and treatment.^1^ It is noteworthy that the absolute number of breast cancer cases is increasing in many developing countries due to population growth and the adoption of western lifestyles.^22^ Fortunately and predictably, the death rates will decline in the future with extending access to advanced prevention, early diagnosis, and medical intervention services for females.^2^

### Breast carcinogenesis and risk factors

Didactically, breast carcinogenesis is a series of genetic and environmental events that drive the multistep process of transformation of normal cells via the steps of hyperplasia, premalignant change, and in situ carcinoma.^23^ Germline mutations and the subsequent second somatic mutation (also known as the “two-hit” model) caused by various environmental factors or exposure to high estrogenic factors lead to the accumulation of genomic changes.^24^ The clonal accumulation of cells leads histologically to ductal hyperplasia, initially without atypia. While in the promotion phase, the expansions of mutation clones form by stimulating the cellular proliferation of autocrine growth factors or recruiting inflammatory and stromal cells to produce these factors, evolving mechanisms to evade the immune system.^25^ These accumulative alterations from both genomic instabilities and external factors result in precursor lesions. Under the long-term action of these carcinogenic alterations, cells continue to adapt and select, and this change gradually increases and accumulates.^26^ DNA damage or mutations develop to a certain extent, which exceeds the limit of self-repair, contributing to in situ carcinoma, where the pathological cells are confined within the ducts but have not yet invaded the surrounding tissues.^27^ The pathological journey of breast cancer from in situ to invasive cancer is another complex process, starting with abnormally proliferating cells in the breast lobules. This transition is characterized by acquiring invasive and metastatic properties, facilitated by genetic alterations and interactions with the tumor microenvironment (Table 1).^28–47^ The invasive phase is often marked by increased aggressiveness and a higher risk of distant spread, underscoring the importance of early detection and interventions.^48^ Hence, breast carcinogenesis is a multistep process involving the accumulation of genetic alterations and the influence of various risk factors.

**Table 1:** Specific genes related to the progression of breast cancer

Genes	Variation	Cellular function and mechanisms of progression	Reference
*NF1*	Mutation	Inhibiting RAS/Raf pathway and promote ER phosphorylation	^28^
*ESR1*	Mutation, copy number variation, gene fusion	Inducing constitutive ER activity, leading to induction of ER target gene transcription that is resistant to endocrine blockade	^29–34^
*ALDH2*	Copy number gain	Copy number gain of ALDH2 are associated with the expansion of subclones with high metastatic potential and shorter patient survival	^35,36^
*GATA3*	Mutation	Negatively regulating the expression of several genes that promote breast cancer metastasis	^37^
*KMT2C*	Mutation	Driving metastasis of TNBC via KDM6A-matrix metalloproteinase 3 axis	^38,39^
*PTEN*	Gene loss	Up regulating CCL2 expression increase the number of CCR2-dependent macrophages, and promote PI3K/ Akt signaling pathway	^40^
*FOXM1*	Upregulate gene expression	Promoting the transcription of G2 and M phase genes in tumor cells through the PI3K/ Akt/mTOR pathway	^41^
*YTHDF3*	Upregulate gene expression	Binding to the m6A methylation site of YTHDF3	^42^
*P53*	Downregulate gene expression	Promoting the secretion of IL-1β by macrophages and the Wnt/β-catenin pathway	^43^
*PI3KCA*	Mutation	Activating the PI3K enzyme and signaling pathway	^44,45^
*RB1*	Mutation	Affecting the CDK-Rb-E2F signaling pathway	^44,46^

*ER* estrogen receptor, *TNBC* triple-negative breast cancer, *ALDH2* aldehyde dehydrogenase 2 family member 2, *KDM6A* lysine specific demethylase 6A, *YTHDF3* YTH domain family 3, *CCL2* C-C Motif Chemokine Ligand 2, *PI3K* phosphoinositide 3-kinase, *RB* Retinoblastoma retinoblastoma gene 1, *CDK* cyclin-dependent kinase, *E2F* E2 factor

**Fig. 2:**
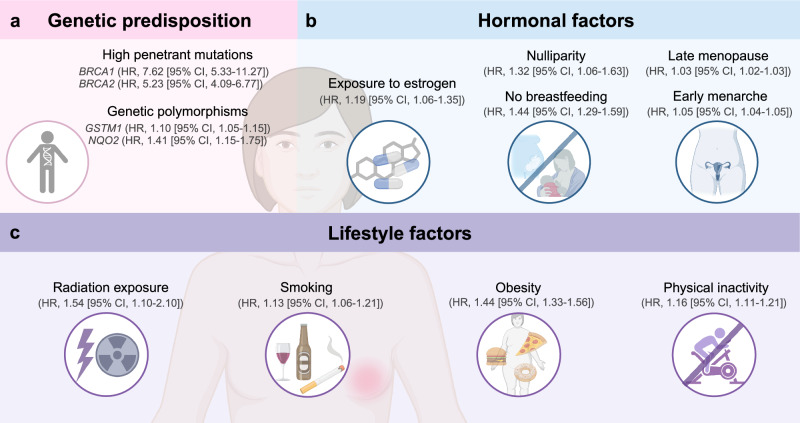
Risk factors for breast cancer. Hormonal factors such as long-term exposure to estrogen, nulliparity and no breastfeeding, late menopause, or early menarche increase the risk of breast cancer. Genetic predisposition is a serious health hazard. High penetrant mutations and genetic polymorphisms are the two parts. Patients with genetic mutations such as *BRCA1*/*2* or patients whose first-degree relative has history of breast cancer are more susceptible to this malignancy. Low penetrant mutations, including *GSTM1* and *NQO2*, are included in genetic polymorphisms of breast cancer susceptibility. Unhealthy lifestyle may also lead to breast cancer. Overdose exposure to radiation and/or heavy alcohol consumption, smoking, having diet high in fat or sugar, obesity, physical inactivity are the leading causes. HR hazard ratio, CI confidence interval. The figure was created with Biorender.com

The leading risk factors involve a combination of genetic predisposition, hormonal factors, reproductive history, and lifestyle choices (Fig. 2).^5^

#### Genetic predisposition

Genetic predisposition is the first and the most noticeable part.^49^ An inherited susceptibility to breast cancer is based on an identified germline mutation in one allele of a moderate to high penetrance susceptibility gene (such as *BRCA1/2*, *CHEK2*, *PALB2*, and *TP53*). Inactivation of the second allele of tumor suppressor genes would be an early event in this oncogenic pathway.^3,50^ Protein-truncating variants in five genes (*ATM*, *BRCA1*/*2*, *CHEK2*, and *PALB2*) were associated with a risk of breast cancer.^6,51^ However, above moderate to high penetrance susceptibility gene mutations only account for ~5% of overall breast cancer cases^3^; attention should be paid to low penetrance susceptibility genetic variation. It mainly includes single-nucleotide polymorphism, insertion/deletion polymorphism, copy number variation, etc. Typical genes, such as *CYP17*, *CYP19*, *GSTM1*, and *NQO2*, are involved in estrogen synthesis.^52–54^ Although the effect of individual sites of these low penetrance genetic variants is weak, the superposition or synergistic effect of multiple sites plays a crucial role in the risk of breast cancer. Notably, the co-occurrence of genomic alterations like *TP53*^mut^-*AURKA*^*amp*^ are deeper insights that reveal the underlying genomic changes in breast cancer.^55^

#### Hormonal factors

Hormonal factors like long-term exposure to estrogen^56^and reproductive history influenced by factors such as late menopause,^57^ early menarche for every year younger at menarche,^58^ nulliparity,^59^ and abortion^60^ exhibit a connection to an elevated vulnerability to breast cancer.^61^ Childbirth and breastfeeding have been shown to mitigate the predisposition to breast cancer, possibly due to hormonal changes and differentiation of breast tissue.^62^

#### Unhealthy lifestyles

Lifestyles cannot be neglected: exposure to radiation,^63^ obesity and physical inactivity,^64^ alcohol consumption,^65^ and smoking^66^ are modifiable risk factors that have been linked to an increased risk of breast cancer.^67,68^ Notably, we have an updated understanding of the risk factors for breast cancer. Circadian rhythm disorder can change the expression of clock genes, disrupt the normal cell cycle, and then directly promote the initiation of malignancy.^69–71^ Indirectly, the disorders probably inhibit melatonin secretion and accelerate the inflammatory response, thus facilitating oncogenesis.^72^ Although the mechanism remains unclear, regular physical activity is regarded as a protective factor of breast cancer incidence.^73^ Several hypotheses aim to explain why physical activity might prevent cancer by reducing the exposure to endogenous sex hormones, altering immune system responses, or insulin-like growth factor-1 levels.^74^

Although risk factors brought about by environmental changes may be an important cause of breast cancer, we cannot ignore the serious consequences of genetic changes interacting with them. Next, we will look at classic examples of gene-environment interactions and offer our views.

### Gene-environment interaction studies

As described above, various factors, the dual interplay between genetic susceptibility and environmental exposures, defined here as radiation, chemicals, and additional external factors, have become a critical area of research on the lengthy issue of breast cancer origin.^75^ Both environmental and genetic factors are critical in a local cellular milieu where tumors initiate and determine the fate of cells.^76^ For the vast majority of diseases, it is apparent that combinations of synergistic or antagonistic factors of both gene and environment are crucial to the risk. Studies have demonstrated environmental factors, such as diet, tobacco intake, chemical exposures, and outdoor light at night, can influence gene expression and contribute to breast cancer risk.^77^ Tobacco smoking increases stop-gain mutations, which may lead to early termination of protein-coding and disrupt the formation of tumor suppressor factors, thereby elevating the susceptibility to breast cancer.^78^ On the flip side, insulin resistance single-nucleotide polymorphisms and lifestyles combined synergistically increased the risk of breast cancer in a gene-behavior, dose-dependent manner, suggesting lifestyle changes can prevent breast cancer in women who carry the risk genotype.^79^ So, it is not hard to imagine that an individual carrying a particular genetic variant may be at greater risk for breast carcinoma if a related pernicious environmental factor is present. Although the potential gene-environment interactions that have been identified are of small to moderate magnitude (probably limited by the number of populations included),^80,81^ we should regard hereditary variants and environmental factors as additive risks in the prediction of breast cancer susceptibility. Studies are focusing on how these exposures interact with genetic factors to affect cancer development, such as multiple metabolic reprogramming and increased susceptibility to breast cancer.^7,82–85^ Currently available research models for studying gene-environment interactions including genome-wide association study, genome-wide interaction search, Bayes model averaging approach, binary regression model, logistic regression model, etc. Knowledge of gene-environment interaction is essential for risk prediction and the identification of specific high-risk populations to inform public health strategies for targeted prevention.

### AI and big data-assisted analysis of individual risks

As the incorporation of AI in disease management covers multiple fields, including screening, diagnosis, relapse forecasting, survival duration prediction, and treatment efficacy measurement, it offers new insights into risk prediction and personalized prevention.^86–88^ Algorithms and models brought by interdisciplinary research, such as deep-learning models, spiking neural networks, deep belief networks, convolutional neural networks, etc.,^89,90^ can analyze vast amounts of data in a shorter time to identify patterns, predict individual risk, and give recommendations more accurately than traditional assays.

For the screening and diagnosis of breast cancer, the convolutional neural network is mainly used for image classification of cancer. It carries out a series of nonlinear transformations on structured data (such as the original pixels of the image) and automatically learns related features of the image, which does not require manual sorting like traditional machine learning models. With the help of the AI, radiologists decrease their false positive rates by 37.3% but maintaining the same level of sensitivity.^91^ AI support systems including Transpara and MammoScreen have been approved by the Food and Drug Administration for clinical practice.^92^ Specifically, AI diffraction analysis is a novel tool for recognizing cells directly from diffraction patterns and classifying breast cancer types using deep-learning-based analysis of sample aspirates for breast cancer diagnosis of fine needle aspirates.^93^ Other AI-based pathological diagnostic tools for breast cancer include slide-DNA, slide-seq, DeepGrade based on digital whole-slide histopathology images contribute to improving the both efficacy and accuracy.^94–96^ As for therapy and drug development, deep-learning algorithms play vital roles in drug screenings.^97^ The AI clinical decision-support systems Watson for Oncology provides individualized evidence-based treatment advice, especially at centers where expert Breast Cancer Resources are limited.^98^

From the above, AI and Big Data-assisted analysis have been shown to give its high inputs in the automated diagnosis as well as treatment of breast cancer, even in managing epidemics, machine learning assists in achieving elementary epidemiological breast cancer prediction by country to exam the emerging risk factors and estimate corresponding incidence rate for a future interval of years.^99,100^ In the near future, these AI-driven strategies will help tailor individual risk profiles and provide targeted prevention.

In short, breast cancer’s multifaceted nature arises from a complex interplay of genetic and environmental factors, leading to various molecular subtypes with distinct pathophysiologies. Understanding these subtypes is crucial for personalized treatment and prognosis.

## Pathophysiology and molecular subtypes of breast cancer

Pathophysiology and molecular subtypes of breast cancer are crucial for understanding the disease’s development, progression, and response to treatment. This knowledge aids in the development of targeted therapies, personalized medicine, and improved patient outcomes. Recognizing subtypes allows for tailored treatment plans, enhancing survival rates and quality of life for those affected by breast cancer.

### Clinical and pathological characteristics of breast cancer

The clinical presentation can vary from a painless palpable breast mass to more advanced symptoms such as skin changes, nipple discharge, or local pain, with or without palpable axillary mass, nipple discharge and inversion, and breast skin thickening.^15^ Patients that are presented as only axillary lymph node metastases (known as occult breast cancer), which account for about 0.3–1.0%,^101^ are easy-to-miss diagnosis and need to be paid more attention. Pathologically, breast cancer is classified into breast invasive carcinoma (70–75%) and lobular carcinoma (12–15%) as suggested by the World Health Organization classification.^102^ There are also eighteen other uncommon subtypes, with a proportion of 0.5–5%.^102^ The pathological descriptions should also include the histological type, histological grade, immunohistochemistry assessment of hormone receptor (HR) status [estrogen receptor (ER) and progesterone receptor (PR) status], human epidermal growth factor receptor-2 (HER2) expression or HER2 gene amplification, and Ki67. For further prognostic evaluation and clinical decision-making, breast cancer can be classified into three subgroups based on immunohistochemical staining results for ER, PR, and HER2: HR-positive/HER2−negative (HR+/HER2−, ~70%), HER2−positive (HER2+, ~15–20%), and triple-negative breast cancer [TNBC, HR-negative (HR-), HER2−, ~15%].^15,103^ Of additional concern, the prevalence of the HR+/HER2− subtype (~50–60%) in China is lower than that in white women, which probably lies in the younger age of the affected population in China, while HER2− subtype accounts for 25% and TNBC accounts for ~15–25%.^104^ In clinical practice, immunohistochemical results are often used to define the four subtypes, namely luminal A, luminal B, HER2−enriched, and TNBC. Luminal A is characterized by high ER and PR and overexpression of the HER2 receptor and Ki67, which indicates slower cell growth, better prognosis, and better response to hormone therapy.While luminal B cancers are also HR-positive but can be either HER2+ or HER2−. They have higher levels of Ki67, indicating faster cell growth and may be treated with hormone therapy and chemotherapy. HER2−enriched ones have high levels of the HER2, which are often more aggressive but can benefit from HER2−targeted therapies. TNBCs do not express ER, PR, or HER2, with a higher risk of recurrence and poorer prognosis. Each subtype has unique clinical outcomes, phenotypes, and therapeutic sensitivities, which guide treatment decisions and influence prognosis.

The precise mechanisms of breast cancer progression are not fully understood. As mentioned above, the etiology of breast cancer involves a complex array of genetic and environmental factors that contribute to the malignant transformation of breast cells. The tumor microenvironment, characterized by interactions between tumor cells, stromal cells, and immune cells, further modulates carcinogenesis. Understanding these mechanisms is vital for developing preventive strategies and targeted therapies.

Extensive research has characterized the molecular features of breast cancer and outlined its progression. At the cellular level, both the clonal evolution model and the cancer stem cell model are widely accepted, with the possibility of tumor stem cells evolving clonally, adding complexity to the situation.^105^ Morphologically, a spectrum of changes and genetic alterations occurs as normal glandular tissue transitions to cancer. Molecularly, numerous gene mutations, hormonal receptor changes, and immune interactions occur throughout the tumorigenesis and progression of breast cancer. The identification of breast cancer susceptibility genes *BRCA1/2*, whose proteins are involved in DNA repair through homologous recombination,^106,107^ has shed light on some mechanisms behind sporadic and hereditary breast cancers. The primary pathogenic mechanism contains genetic alternations, hormonal homeostasis changes, and immune interference, which are demonstrated as follows.

#### Genetic alterations

Genetic mutations are the basis for carcinogenesis. Carrying the heterozygous mutation of *BRCA1/2*, transformation to complete malignancy of cells occurs after a serious external secondary hit, further resulting in genome instability and cellular disorders. The genetic instability ulteriorly leads to genetic alterations in cells, such as somatic mutations of *PIK3CA* and *TP53*, which are non-inherited.^108^ Additionally, chromosomal instability, which is a hallmark of cancer,^109^ is responsible for driving somatic copy number variations and intratumor heterogeneity within subclones during cancer progressions.^110^ In the process of tumor evolution, DNA copy number loss, transcript repression, epigenetic silencing, and whole-genome doubling are different ways for potential malignant cells to acquire immune evasion and fueling adaptive abilities in response to various pressure.^110,111^ Through a series of complex disruptions of the genome, cells acquire the accumulation of deleterious alterations irreversibly to survive in purifying selection (removing deleterious genetic variations) of human germline evolution, further obtaining fitness, attenuating tumor cell attrition and evolving to malignancies.^112^

#### Changes in hormonal homeostasis

Hormonal exposure (including menopausal hormone therapy, overdose estrogen intake from food, and endocrine instability caused by various reasons) accounts for the main contributing factors for sporadic breast malignancies. Specifically, estrogen binding to the nuclear ER (encoded by *ESR1*) is an inducer of breast cancer. Imbalances between estrogen and progesterone can promote cell proliferation and potentially lead to the accumulation of DNA damage. At this juncture, excess estrogen promotes the expansion of these malignant cells and triggers an increase in the supportive stroma, which in turn facilitates the progression of cancer.^113^ Upon ligand engagement, the ER modulates the transcription of genes by binding to estrogen response elements in their promoter areas, thereby controlling gene expression.^114^ Additionally, ER can engage directly with other proteins, including those involved in growth signaling pathways, which, in turn, amplifies the transcription of genes that are pivotal for cellular expansion and resistance to apoptosis.^115^ In a word, disturbances in estrogen homeostasis in the breast tissue may promote breast cancer progression and metastasis.

#### Immune interference

Breast cancer cells develop within a complex microenvironment that includes various benign cell types and extracellular matrix. Cancer-associated fibroblasts (CAFs) are the predominant cell type present; however, the breast cancer microenvironment is also populated by lymphocytes, macrophages, myeloid lineage cells, etc., which predominantly play roles in immune reactions.^116,117^ In the early stages of tumor development, the immune microenvironment mainly suppresses tumor proliferation through the cytokine environment produced by activated CD8+ and CD4+ T cells. Whereas, once the tumor turns aggressive, tumor cells express immune checkpoint modulators [such as cytotoxic T lymphocyte-associated protein 4 (CTLA-4) and programmed cell death 1 ligand 1 (PD-L1)] to suppress the immune response. The composition of microenvironmental cells, including CAFs and the content of cytokines, is influenced by the “invasion” of breast cancer cells, thereby promoting tumor progression.^118^

Breast cancer exhibits unique mechanisms of immune evasion that contribute to its progression and resistance to immunotherapy. Breast cancer can evolve over time, leading to increased genomic complexity and heterogeneity, which may impose selective pressures and result in differential responses to therapies.^119^ Detailly, breast cancer cells mimic the anti-inflammatory mechanism of central nervous system to evade antitumor immunity, which is dependent on the immunological synapse.^120^ Carrying lower clonal heterogeneity and neoantigen loads, TNBC cells achieve immune escape via Lgals2-CSF1-CSF1R axis,^121^ which is also a specific mechanism in breast cancer immune escape.

The interplay between breast cancer cells and host antitumor immunity determines co-existing mechanisms of immune escape within the same patient, highlighting the need for combinatory immunotherapies and biomarker development.

### Molecular subtypes and variability in tumorigenesis across different subtypes of breast cancer

Understanding the molecular subtypes and variability in tumorigenesis across different breast cancer subtypes allows for tailored treatments, improved patient outcomes, and the discovery of new therapeutic targets. This understanding is critical for advancing clinical trials and translating research into clinical practice, ultimately revolutionizing breast cancer management.

#### Variability in tumorigenesis across different subtypes

As mentioned in the previous part, the immunogenicity of breast cancer varies among multiple molecular variants, with TNBC and HER2+ tumors being more immunogenic, while luminal A and luminal B subtypes are less immunogenic.^119^ Since breast cancer is a highly heterogeneous disorder, it is not surprising that the subtype changes metastasis or under the pressure of therapies.^122^ Neoadjuvant chemotherapy can probably change ER and PR expression and status. Changes in ER, PR, and HER2 receptors are more evident in patients treated with neoadjuvant chemotherapy and trastuzumab than those without. It is worth noting that retesting of the hormone and HER2 receptors should be considered in certain situations to optimize adjuvant systemic therapy.^123^

#### Molecular subtypes of breast cancer

**Fig. 3:**
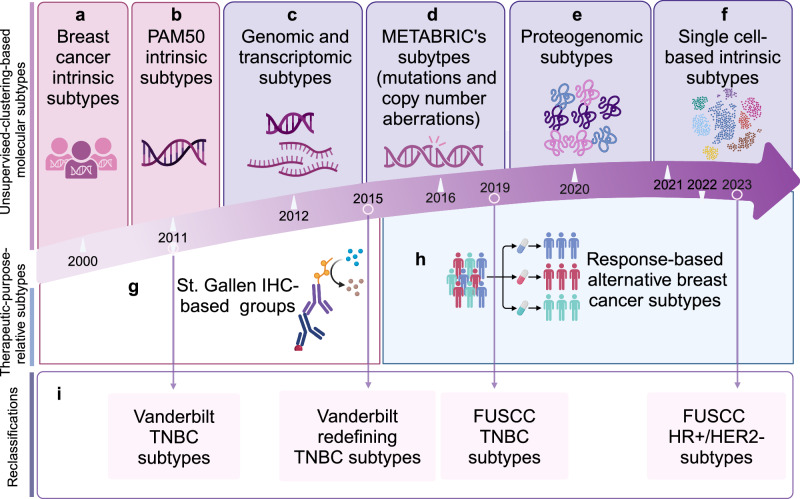
General timeline for redefining breast cancer molecular subtypes. The subtypes of breast cancer can be divided into two groups, namely unsupervised-clustering-based molecular subtypes and therapeutic-purpose-relative subtypes. **a** Perou et al. firstly proposed the concept of molecular typing of breast cancer in 2000 by using DNA microarrays representing >8000 genes. **b** Parker et al. constructed PAM50 subtypes in 2009, which was a simplified version of the “intrinsic” subtypes. **c** In 2012, Christina et al. offered an integration of the genome and transcriptome from representative patients, which provided a novel molecular stratification of the breast cancer population. **d** Bernard et al. identified ten subtypes of breast cancer from the landscape of mutations, driver copy number aberrations. **e** Unsupervised proteogenomics identified four molecular subtypes underscore the potential of proteomics for clinical investigation in 2020. **f** In 2021, another update called single-cell method of intrinsic subtype stratified the complex cellular ecosystems into nine clusters. **g** IHC-based subtype was the first therapeutic-purpose-relative subtype raised in 2011. **h** An alternative subtype was constructed in 2022 according to various regimens redefined and supported the usage of response-based subtypes to guide future treatment prioritization. **i** Reclassifications of the specific subtypes include Vanderbilt TNBC subtypes in 2011, Vanderbilt redefining TNBC subtypes in 2015, FUSCC TNBC subtypes in 2019 and FUSCC HR+/HER2− subtypes in 2023. IHC immunohistochemistry, TNBC triple-negative breast cancer, FUSCC Fudan University Shanghai Cancer Center, HR hormone receptor, HER2 human epidermal growth factor receptor-2. The figure was created with Biorender.com

The past few decades have witnessed the promotion and popularization of the concept of classification-based treatment (Fig. 3). Roughly, the subtypes of breast cancer can be divided into two groups, namely unsupervised-clustering-based molecular subtypes and subtypes with therapeutic intent. With the development of sequencing techniques, the unsupervised-clustering-based molecular subtypes have been iteratively updated substantially. In 2000, the concept of molecular typing of breast cancer was born.^124^ According to the similarities and differences of gene expression profiles, tumors can be divided into luminal A/B, HER2−enriched, basal-like, and normal-like subtypes. In 2009, PAM50 assay redefined those subtypes using the microarrays of fifty genes.^125^ In 2012, integrating the genome and transcriptome from representative patients provided a novel molecular stratification of the breast cancer population.^126^ This unsupervised analysis revealed novel subgroups with distinct clinical outcomes, which reproduced in the validation cohort. Deletions in PPP2R2A, MTAP and MAP2K4 were identified by delineating expression outlier genes driven in cis by CNAs. In 2021, the complex cellular ecosystems were stratified into nine clusters according to a single-cell method of intrinsic subtype,^127^ which broadened our horizons of our limited understanding of cellular composition and organization in breast cancer. In this classification, the stromal-immune niches were spatially organized in tumors, offering insights into antitumor immune regulation. After the overall classification, reclassification after the general subtype also emerged in an endless stream. Studies have shown that TNBC is a group of diseases with molecular genetic heterogeneity. Lehmann et al. divided TNBC into six subtypes from the molecular classification: basal-like 1/2, immune modulative, mesenchymal, mesenchymal stem cell-like, and luminal androgen receptor subtypes,^128^and subsequently Burstein et al. refined the six TNBC subtypes into four subgroups.^129^ Based on the cohort from Fudan University Shanghai Cancer Center, molecular classifications of TNBC and HR+/HER2− breast cancer were further developed.^130–132^

As for the therapeutic-purpose-relative subtype, St. Gallen International Breast Cancer Conference adopted an immunohistochemical-based subtype including luminal A-like, luminal B-like, HER2 overexpression (non-luminal), and basal-like subtypes.^133^ Subsequent studies showed significant differences in breast cancer prognosis among different molecular subtypes.^134^ In 2022, an alternative subtype was constructed according to various regimens redefined and supported the usage of response-based subtypes to guide future treatment prioritization.^135^ More than 11 subtyping schemas were explored and this redefinition identified treatment-subtype pairs maximizing the pathologic complete response (pCR) rate over the population. Understanding these subtypes is critical for the development of targeted therapies and personalized medicine approaches.

Taken together, the clinical and pathological characteristics, pathogenic mechanisms, and molecular subtypes of breast cancer collectively contribute to its complexity and diversity. Continued research in these areas is essential for enhancing the precision of diagnoses, optimizing therapeutic approaches, and boosting patients’ survival.

## Mechanisms of breast cancer progression: frontier research

**Fig. 4:**
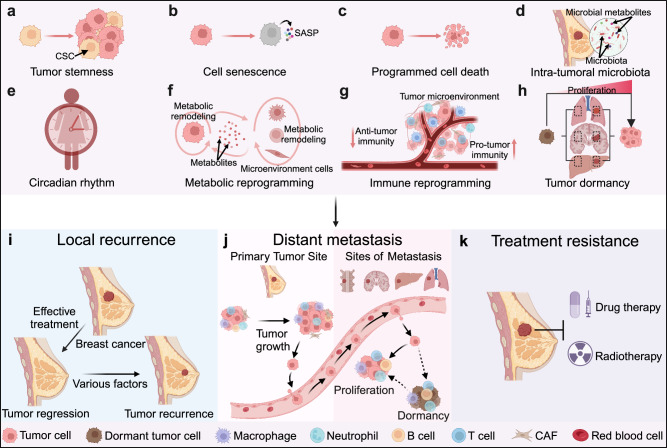
Diverse factors regulating the progression of breast cancer. Many factors contribute to the progression of breast cancer, resulting in local recurrence (**i**), metastasis (**j**), and treatment resistance (**k**) of breast cancer. These factors include tumor stemness (**a**), cellular senescence (**b**), novel types of programmed cell death (**c**), intra-tumoral microbiota (**d**), circadian rhythm (**e**), metabolic reprogramming (**f**), immune reprogramming (**g**), as well as tumor dormancy (**h**). CSC cancer stem cell, SASP senescence-associated secretory phenotype. The figure was created with Biorender.com

For all tumors, including breast cancer, tumor progression results in local relapse, metastasis, and treatment resistance, and represents great clinical challenges that need to be addressed.^8,136,137^ With the continuous advancements of experimental techniques and sequencing technology (such as single-cell sequencing and spatial omics), significant strides have been made in comprehending the underlying mechanisms driving tumor progression. Belows are highlighted some key factors contributing to the progression of breast cancer (Fig. 4).

### Tumor stemness

Cancer stem cells (CSCs) constitute a minor fraction of the tumor population, characterized by their capacity for self-renewal and differentiation.^138,139^ A plethora of compelling evidence substantiates that CSCs play a pivotal role in driving tumor initiation, conferring resistance to treatment, facilitating recurrence, and promoting metastasis.^140,141^ Although CSCs represent a functional cellular state, it has been demonstrated that their identification can be facilitated by utilizing specific cell markers such as CD133, CD44, EPCAM, and ALDH1, among others.^142^

In solid tumors, the first identification and isolation of CSCs was conducted in breast cancer,^143^ which also plays a significant role in its progression. Kita-Kyushu lung cancer antigen-1 (KK-LC-1), identified as a novel marker for TNBC CSCs, inhibits Hippo signaling by binding to FAT1. This facilitates the nuclear translocation of YAP1, subsequently triggering the transcription of *ALDH1A1*. Pharmacological inhibition of downstream signal transduction mediated by KK-LC-1 significantly impairs TNBC tumor growth.^144^ EMSY is also a newly discovered biomarker of TNBC CSCs, It competitively binds to the Jmjc domain, which is critical for KDM5B enzyme activity, thereby reshaping methionine metabolism in CSCs. This metabolic reprogramming enhances CSCs self-renewal and tumorigenesis through an H3K4 methylation-dependent mechanism.^145^ F-box protein FBXL2, known as a negative regulator of stemness by targeting the transcription factor E47 for polyubiquitination and proteasome-mediated degradation in breast cancer, is significantly down-regulated in paclitaxel-resistant TNBC cells; however, its activator can be utilized to reduce the stemness of TNBC cells and enhance treatment sensitivity to paclitaxel.^146^

An interesting phenomenon is that breast cancer stem cells (BCSCs)—secreted DKK1 can effectively suppress the proportion of the stem cell population both in vivo and in vitro. This reduces tumor initiation ability while simultaneously increasing the expression of solute carrier family 7 member 11 (SLC7A11), which protects tumor cells from lipid peroxidation and ferroptosis that promote tumor metastasis.^147^ Nuclear mRNA export is a crucial step in eukaryotic gene expression. Prostate cancer-associated transcript 6 (PCAT6), a long non-coding RNA, enhances nuclear mRNA export related to BCSCs, thereby increasing stemness and resistance to doxorubicin in breast cancer.^148^ The utilization of radiotherapy is considered a crucial therapeutic modality for treating tumors. However, the emergence of radiation resistance frequently poses significant clinical challenges.^149^ Upregulation of THOC2 and THOC5 protein expression can promote the THOC-mediated spliced mRNA efflux, leading to increased synthesis of NANOG and SOX2 proteins; This process can strengthen the stem-like properties of TNBC cells and contribute to their increased resistance to radiotherapy.^150^

The stemness of tumor cells is regulated by internal signals and influenced by the tumor microenvironment. LSECtin, a transmembrane protein expressed primarily on macrophages, can enhance breast cancer stemness and promote the growth of breast cancer in a contact-dependent manner.^151^ Moreover, breast cancer cells can secrete CCL20 to stimulate the production of a significant amount of C-X-C motif chemokine ligand 2 (CXCL2) by polymorphonuclear myeloid-derived suppressor cells. The binding of CXCL2 to the CXCR2 on the surface of tumor cells activates the CXCR2/NOTCH1/HEY1 signaling pathway, leading to increased tumor cell stemness and mediating resistance to docetaxel.^152^ In addition to immune cells, TNBC tumor cells also receive secretory signals from CAFs through the IL-8/CXCR1/2 axis to maintain their stemness state.^153^ The extracellular matrix (ECM), a non-cellular component of the tumor microenvironment, has a physical structure and chemical composition often associated with tumor progression.^154^ The relatively low mechanical stress (about 45 Pa) derived by the ECM can stimulate the stem cell signaling pathway via the cytoskeleton/AIRE axis by activating the integrin beta 1/3 receptor, while excessive mechanical stress (~450 Pa) induces quiescence in BCSCs dependent on DDR2/STAT1/P27 signaling, which may explain conflicting results observed in previous individual studies.^155^

### Cellular senescence

Cellular senescence is a self-defense mechanism triggered by internal and external stimulation, playing a pivotal role in organismal development and post-injury repair.^156^ Cell cycle arrest, resistance to apoptosis, and senescence-associated secretory phenotype (SASP) are the primary hallmarks of cellular senescence.^157^ It should be noted that cellular senescence should not be conflated with the broader concept of aging, as the latter encompasses a more comprehensive range of phenomena beyond just cellular senescence.^158^ Cellular senescence is intricately involved in various physiological and pathological processes within the body,^159^ exhibiting a dualistic role in tumorigenesis by both promoting and suppressing cancer.^160,161^

In the oncogene-driven Neu and MMTV-PyMT mouse models, the overactivation of the RANK signaling pathway in normal mammary epithelium induces cellular senescence, thereby delaying the onset of breast cancer but promoting subsequent metastatic invasion.^162^ During breast cancer chemotherapy, specific tumor cells exhibit upregulation of SASP genes, accompanied by an augmented expression of immunosuppressive molecules PD-L1 and CD80 within these tumor cells. This phenomenon facilitates immune evasiveness, thereby facilitating the survival of tumor cells during chemotherapy.^163^

The concept of cellular senescence within tumors extends beyond the tumor cells themselves. CAFs are stromal cells within the tumor microenvironment that exhibit diverse biological characteristics, often demonstrating tumor-promoting activity.^164^ The extracellular matrix secreted by senescent CAFs, as identified through single-cell RNA sequencing (scRNA-seq), is found to specifically limit the cytotoxicity of natural killer (NK) cells, thereby promoting tumor growth.^165^ In another study, a distinct senescence-like tetraspanin-8 (TSPAN8)+myofibroblastic CAF (myCAF) subgroup potentiates tumor stemness through SASP-associated factors IL-6 and IL-8, thereby promoting chemotherapy resistance in breast cancer.^166^ Within the tumor microenvironment, neutrophils exist in various functional states that promote or suppress cancer progression.^167^ The breast cancer cells can engulf exosomes secreted by senescent neutrophils, thereby enhancing their resistance to chemotherapy.^168^ Additionally, senescent neutrophils can accumulate at pre-metastatic sites for lung metastasis in breast cancer patients, forming neutrophil extracellular traps that effectively ensnare tumor cells and facilitate lung metastasis.^169^

### Novel types of programmed cell death

Cell death is crucial for the development and maintenance of homeostasis in organisms and can be categorized into accidental cell death and regulated cell death (RCD), depending on its controlled nature.^170^ RCD, also referred to as programmed cell death (PCD), encompasses various forms, including apoptosis, necroptosis, pyroptosis, ferroptosis, entotic cell death, netotic cell death, parthanatos, lysosome-dependent cell death, autophagy-dependent cell death, alkaliptosis, and oxeiptosis; all of which occur through distinct mechanisms and are associated with tumorigenesis and tumor progression.^170–172^

Ferroptosis is a natural antitumor mechanism; however, tumor cells possess a distinct advantage in evading ferroptosis. The occurrence of ferroptosis not only impacts tumor cells but also regulates the antitumor immune response.^173^ Among the four subtypes of TNBC,^132^ the luminal androgen receptor (LAR) subtype exhibits the highest ferroptosis activity. The utilization of glutathione peroxidase 4 (GPX4, one of the core regulatory factors of ferroptosis^174^) inhibitors can effectively attenuate tumor proliferation and enhance antitumor immunity, while combining GPX4 inhibitors with immunotherapy can further impede tumor progression.^175^ Furthermore, in breast cancer, tumors exhibiting high levels of imaging tumor heterogeneity are associated with a poor prognosis. However, these tumors show increased activation of key pathways that promote and inhibit ferroptosis, suggesting that targeting drugs that promote ferroptosis could be an effective clinical target.^176^

Cuproptosis^177^ and disulfidoptosis^178^ have expanded the concept of PCD in recent years. Copper is an indispensable trace element in the human body, and the copper-dependent growth and proliferation of cells are related to various biological behaviors of tumors.^179^ However, excessive intracellular copper accumulation can induce mitochondrial proteotoxic stress and ultimately result in cellular apoptosis, known as cuproptosis, a process primarily regulated by ferredoxin 1.^177^ Targeting cuproptosis holds significant implications for tumor therapy.^180^ In preclinical models of breast cancer, some novel nanomedicines that target cuproptosis have demonstrated efficacy in inhibiting tumor growth and may hold potential for future clinical applications.^181,182^ The occurrence of disulfidptosis is primarily attributed to the elevated expression level of SLC7A11 in cells, which leads to excessive cystine uptake. The metabolism of cystine necessitates the consumption of NADPH. However, inadequate NADPH production in cells with restricted glucose intake significantly augments actin cytoskeletal disulfide bonds, causing disruption in the actin network and intracellular disulfide hyperplasia. Consequently, cell disulfide stress ensues, ultimately leading to cell death.^178^ Although limited research has been conducted on disulfidptosis in tumors, targeting disulfidptosis, such as through glucose transporter inhibitors, could present a novel therapeutic approach for SLC7A11 overexpressing tumors in the future.^183,184^ Both ferroptosis and disulfidptosis depend on intracellular redox homeostasis.^173^ Drugs that disrupt this homeostasis can promote the simultaneous occurrence of both processes, thereby inhibiting tumor progression.^185^ This indicates that these drugs may represent more effective clinical therapeutic targets, warranting further investigation into their potential applications.

### Intra-tumoral microbiota

Although most normal tissues in the human body are commonly perceived as sterile, bacteria^186^ and fungi^187–189^ can be detected in tumor tissues, particularly in tumor cells and immune cells, using various technical methods. These microorganisms are not mere bystanders in the tumor microenvironment; instead, they can promote tumorigenesis and tumor progression.^190–195^

Bacteria within breast cancer cells have been observed in situ tumors.^186^ It has been observed that genera under Clostridiales are enriched in immunomodulatory subtype among TNBC patients, with high levels of its associated metabolite trimethylamine N-oxide (TMAO). TMAO induces pyroptosis of tumor cells by activating the endoplasmic reticulum stress kinase PERK, thus enhancing the antitumor immune effect of CD8+ T cells.^196^ When tumor cells metastasize to distant sites through the circulatory system, they are exposed to severe stress within the blood vessels, such as hemodynamic shear forces and attacks of the immune system.^197^ Remarkably, circulating tumor cells can carry bacteria, which promote cytoskeletal reorganization and enhance the tumor cells’ resistance to fluid shear stress in the bloodstream. This ultimately facilitates host cell survival and distant metastasis.^198^

The origin of microbes in breast cancer has remained an unresolved question. Fusobacterium nucleatum, a bacterium closely associated with colorectal cancer,^192,199^ can translocate to breast cancer tissues exhibiting high Gal-GalNAc expression through hematogenous spread, primarily via the interaction between Fap2 expressed by Fusobacterium nucleatum and Gal-GalNAc on the surface of breast cancer cells. The inoculation of Fusobacterium nucleatum hampers T-cell infiltration within the tumor, thereby facilitating tumor progression and metastasis.^200^

### Circadian rhythm

Life activities follow a 24-hour cycle known as the circadian rhythm or biological clock, which is regulated by intricate signaling pathways within the body and influenced by external factors such as light and temperature.^201,202^ The circadian rhythm exerts an influence on the immune function^203^ and metabolic activities^204^ of the body, playing a pivotal role in upholding normal physiological functions. The disruption of this rhythm is closely linked to a range of diseases, such as neurodegenerative disorders,^205^ cardiovascular diseases,^206^ kidney diseases,^207^ and tumors.^208–212^

Disruptions to the normal circadian rhythm can increase the risk of breast cancer.^213,214^ Specifically, disturbances in the circadian rhythm not only enhance the malignant potential of breast cancer cells (including their ability for self-renewal, replication, metastasis, and invasion) but also impact chemokine/chemokine receptor signaling (the CXCL12-CXCR4 axis may be the primary signaling pathway) which contributes to the formation of an immunosuppressive microenvironment ultimately leading to tumor progression. The CXCR2 chemokine receptor inhibitor can correct the effects of long-term circadian rhythm disruption on the dissemination and metastasis of breast cancer cells.^215^ Furthermore, CTCs are pivotal in tumor dissemination through the bloodstream.^216^ The production of highly metastatic CTCs in breast cancer is significantly higher during sleep compared to the less metastatic CTCs produced during daytime activity, indicating the importance of considering time nodes in clinical sample collection and tumor treatment. Mechanistically, CTCs exhibit high expression of various circadian rhythm hormones receptors, and circadian hormones, such as melatonin, can influence the production of CTCs. Analysis of CTCs obtained from patients and mouse models during the resting phase using scRNA-seq reveals significant upregulation of mitotic genes, which may contribute to the enhanced metastatic potential of CTCs.^69^

### Metabolic reprogramming

One hallmark of tumors is altered energy metabolism, with the most well-known example being the Warburg effect.^217^ Throughout the progression from precancerous lesions to localized tumors and from tumors in situ to metastatic tumors, the metabolic preferences of tumor cells continuously change in response to cellular states and environmental conditions, which are regulated by endogenous signals from tumor cells and signals from the tumor microenvironment.^218^ The alterations in tumor cells’ metabolic preferences are associated with tumor progression.^219–221^

*MYC* is a commonly occurring oncogene,^222^ yet the metabolic characteristics of tumors with high *MYC* expression are still worth exploring. In breast cancer, MYC regulates the elevated expression of vitamin transporter SLC5A6 in tumor cells, promoting intracellular transport of vitamin B5 and its conversion to coenzyme A. This enhances metabolic pathways such as the tricarboxylic acid cycle and fatty acid biosynthesis, ultimately supporting tumor growth.^223^ Dynamin-related protein 1 promotes fragmented mitochondrial puncta formation in latent brain metastatic cells, leading to a shift towards fatty acid oxidation (FAO) metabolism that maintains redox homeostasis and survival of tumor cells within the brain microenvironment.^224^ Analysis of scRNA-seq and spatial transcriptomics data from paired primary breast cancer tumors and lymph node metastatic tissues revealed that the process of lymph node metastasis in breast cancer involves a metabolic shift from glycolysis to oxidative phosphorylation and back to glycolysis, indicating a potential target for the treatment of tumor metastasis.^225^

The altered metabolic characteristics of brain metastatic cells in breast cancer not only affect tumor cells themselves but also impact antitumor immunity within the brain microenvironment. In HER2+ breast cancer, the metabolic characteristics of synchronous brain metastatic (S-BM) cells, metachronous brain metastatic (M-BM) cells, and latent (Lat) brain metastatic cells are distinct. S-BM cells exhibit increased glycolytic activity, resulting in elevated lactate production. This lactate, secreted into the tumor microenvironment, inhibits the function of NK cells, aiding tumor cells in evading immune surveillance. Inhibiting lactate metabolism in S-BM cells significantly impedes metastasis. M-BM and Lat cells demonstrate enhanced capabilities to utilize glutamine in response to oxidative stress due to the high expression of the anionic amino acid transporter (xCT), which enhances the survival capacity of tumor cells. Pharmacological inhibition of xCT can reduce residual disease and recurrence.^226^

Notably, the metabolic characteristics of non-tumor cells within the tumor microenvironment also undergo alterations to support tumor growth and metastasis.^227–230^ CAFs enhance their glycolytic activity, producing large amounts of lactate that breast cancer cells can absorb and utilize.^231^ Resident lung mesenchymal cells accumulate neutral lipids intracellularly during the pre-metastatic breast cancer lung metastasis phase. These lipids are transferred via vesicles to tumor cells and NK cells, promoting tumor cell proliferation while inhibiting NK cell function.^232^ During breast cancer progression, the stiffened fibrotic tumor microenvironment enhances the TGFβ autocrine pathway in tumor-associated macrophages (TAMs), activating their collagen biosynthesis program. This process consumes large amounts of arginine and increases ornithine secretion. Reduced arginine and elevated ornithine levels in the tumor microenvironment impair CD8+ T-cell function, ultimately leading to tumor progression.^233^

### Immune reprogramming

A properly functioning immune system is crucial for killing and eliminating tumor cells.^234–237^ Unfortunately, tumor cells often “remodel” the tumor immune microenvironment through various mechanisms to achieve immune evasiveness,^238^ such as reducing tumor antigen presentation, decreasing the infiltration or function of tumor-inhibitory immune cells, and promoting the infiltration of immunosuppressive cells.^239,240^

The precise mechanisms by which tumor cells restrict immune cell infiltration into tumors remain incompletely understood. In PTEN-deficient breast cancer, the expression of PI3Kβ in tumor cells significantly hampers the infiltration of CD4+ and CD8+ T cells via the BMX/STAT3 signaling pathway, leading to the formation of an “immune desert” within tumors and facilitating tumor immune evasiveness.^241^ Another study indicates that the extracellular domain (ICD) of discoidin domain receptor 1 is released by tumor cells during tumor progression, causing changes in the alignment of collagen fibers in the extracellular matrix (ECM). This alteration forms a barrier to immune cell infiltration, protecting tumor cells from immune cell-mediated killing.^242^

The function of immune cells infiltrating tumor cells can also be suppressed, preventing them from exerting their typical effects. In immune checkpoint inhibitor-resistant HER2+ breast cancer, tumor cells upregulate the expression of N-acetyltransferase 8-like to produce high N-acetylaspartate (NAA) levels. After being absorbed by NK cells and CD8+ T cells, NAA can inhibit the formation of immunological synapses in these cells, leading to immune evasiveness.^120^ Additionally, FGF21 secreted by breast cancer cells can alter cholesterol metabolism in CD8+ T cells, causing excessive cholesterol biosynthesis and inducing CD8+ T-cell exhaustion.^243^ The TAMs are immune cells that exhibit immunosuppressive functions, and reducing TAMs infiltration can inhibit tumor growth and improve survival rate.^244^ Using large-scale in vivo CRISPR screening technology, researchers have identified the E3 ligase Cop1 within breast cancer cells as an essential regulator of macrophage chemokine secretion. The expression of Cop1 promotes the secretion of macrophage-associated chemokines by tumor cells, which enhances macrophage infiltration within the tumor, particularly M2 macrophages. Inhibiting Cop1 can enhance antitumor immunity and improves the response to anti-PD-1 therapy.^245^

Before lung metastasis in breast cancer, there was a discernible alteration in the local pulmonary microenvironment, primarily characterized by a decrease in the quantity and impaired functionality of cytotoxic T lymphocytes and NK cells, potentially mediated by the primary tumor.^246^ In comparison to the primary site, breast cancer metastases (including lymph nodes, lung, liver, and brain) also undergo substantial immune reprogramming with an augmented presence of immunosuppressive cells and compromised antitumor immunity.^246–248^

The functions of B cells are diverse and play a crucial and complex role in tumor progression.^249^ In patients with TNBC who have received neoadjuvant chemotherapy, researchers utilized scRNA-seq to identify that chemotherapy can induce the accumulation of ICOSL+ B cells. Specifically, complement signals triggered by chemotherapy-induced immunogenic cell death of tumor cells promote the transition of other B cells into ICOSL + B cells. These B cells promote T-cell-dependent antitumor immunity, thereby enhancing the efficacy of chemotherapy.^250^ Additionally, Furthermore, pathological antibodies secreted by B cells bind to the HSPA4 receptor on the surface of tumor cell membranes, thereby initiating downstream signaling pathways that activate the NF-κB pathway in tumor cells. This activation results in the expression of target genes HIF1α and COX2. The former promotes the expression of the chemokine receptor CXCR4 in tumor cells, while the latter induces lymph node stromal cells to secrete the chemokine SDF1α. Consequently, this process fosters the formation of a pre-metastatic microenvironment and directs tumor cells toward the draining lymph nodes.^251^

### Tumor dormancy and reactivation

The dormancy and reactivation of long-established disseminated tumor cells (DTCs) in distant organs following primary tumor resection constitute a pivotal factor contributing to tumor recurrence and pose a significant challenge in antitumor therapy.^136,252–254^ Breast cancer continues to recur 5-20 years post-treatment, particularly in ER+ breast cancer cases.^255,256^ The implication is that the distal site DTCs have remained dormant for numerous years before the clinical detectability of the tumor.^257^

Previous investigations have elucidated specific mechanisms underlying the dormancy and reactivation of breast cancer cells,^258–262^ while recent discoveries have further enhanced our comprehension of these phenomena. Type III collagen secreted by breast cancer cells can act as a pivotal “switch” in the ECM. When present in abundance, it sustain tumor dormancy, as its disruption promotes tumor cell proliferation through DDR1-mediated STAT1 signaling.^263^ Researchers have discovered that NK cells maintain the dormant state of tumor cells within the liver. Excessive accumulation of activated hepatic stellate cells inhibits the proliferation of NK cells, resulting in the activation of tumor cells and subsequent macroscopic liver metastasis.^264^ Moreover, in the liver metastasis model of breast cancer, the interaction between NK cells and activated hepatic stellate cells (aHSCs) also serves as one of the “switch” of tumor dormancy. On one hand, IFN-γ secreted by NK cells sustains tumor dormancy. On the other hand, the chemokine CXCL12 secreted by aHSCs can induce the quiescent state of NK cells through its homologous receptor CXCR4, thereby triggering the activation of tumor cells. In the breast cancer lung metastasis model, the platelet-derived growth factor C (PDGF-C) level in the microenvironment increases when lung tissue becomes senescent or fibrotic. PDGF-C activates fibroblasts, reactivating dormant breast cancer cells in the lung and thereby accelerating metastasis formation.^265^

Furthermore, by regulating stem cell properties in breast cancer cells, long non-coding RNA NR2F1-AS1 facilitates local diffusion while inhibiting lung metastasis activation, ultimately promoting dormancy among breast cancer cells during metastasis.^266^ When breast cancer metastasizes to the brain, DTCs are located on the endfeet of astrocytes. At these sites, laminin-211 secreted from astrocytes binds to dystroglycan, a non-integrin receptor encoded by *DAG1* on the surface of DTCs, promoting DTCs quiescence.^267^ Metabolism is closely linked to tumor dormancy. High levels of the transcription factor *NRF2* can induce metabolic reprogramming in dormant tumor cells, re-establishing redox homeostasis and de novo synthesis of nucleotides, accelerating the activation of tumor cells and tumor recurrence.^268^ The specific mechanisms underlying early occult metastasis in breast cancer remain unknown. The primed pluripotency transcription factor *ZFP281*, regulated by FGF2 and TWIST1, is a key factor in the dissemination and dormancy of early DTCs. ZFP281 inhibits the proliferation of primary breast cancer but drives the epithelial-mesenchymal transition process, thus promoting metastasis. Once tumor cells reach distant metastatic sites, ZFP281 maintains tumor dormancy and prevents tumor proliferation over extended periods via the induction of the class IIcadherin 11.^269^ Unlike the classical view of the metastasis cascade model, this reveals a novel mechanism of metastatic dormancy.

## Diagnosis of breast cancer: technological advancements

With the continuous emergence of new technologies, the diagnosis of breast cancer has gradually moved from the traditional imaging era to the new era of AI, slice multiple staining, and so on. Here we introduced the role of new technologies in the diagnosis of breast cancer in recent years.

### Conventional diagnosis of breast cancer

The routine diagnosis of breast cancer primarily involves imaging examinations, pathological examinations, and clinical physical examinations. The objects of clinical physical examination include the breast, regional lymph nodes, and distant metastases. Imaging examinations include bilateral mammography and ultrasound examination of the breast and regional lymph nodes, while magnetic resonance imaging (MRI) is not routinely recommended.^270,271^

### High-throughput screening technologies

Despite the power of conventional diagnosis technologies, it should be noted that there is a disease case set called occult breast cancer, which cannot be detected by imaging. ctDNA, which is derived from the release of tumor cells,^272^ is a part of the cfDNA library released after cell apoptosis or necrosis. In recent years, the wide application of high-throughput analysis technology has made ctDNA a promising biomarker for screening and diagnosis of breast cancer.^273–277^

Recently, researchers have carefully studied the power of ctDNA to diagnose breast cancer. A meta-analysis that included 24 studies indicated that the average sensitivity and specificity of cfDNA as a diagnostic tool were 70% and 90%,^278^ respectively. Another more comprehensive meta-analysis, which included 29 studies, indicated that the sensitivity and specificity reached 80% and 88%, respectively.^279^ These data confirmed the powerful ability of cfDNA/ctDNA as a diagnostic tool for primary breast cancer.

The diagnosis of advanced breast cancer is also important. In one study, a significant increase in the ctDNA portion was observed 12 weeks before the clinical progression of breast cancer leptomeningeal metastasis (BCLM).^280^ Another study on BCLM showed that the quantification of ctDNA in the participants’ cerebrospinal fluid achieved a remarkable 100% sensitivity and specificity in diagnosing BCLM, exceeding the traditional “gold standard” cytology method.^281^ These data emphasize the critical role of ctDNA in the diagnosis of advanced breast cancer, especially in the case of meningeal metastasis.

### Digital pathology and AI-assisted diagnosis

Digital pathology makes analyzing data from pathological samples easier and provides a deeper understanding of the collected data. Digital methods have higher efficiency in collecting, integrating, and analyzing data than traditional technologies. It has excellent potential to achieve more reliable and accurate data processing for data of larger scale.^282^ Multi-omics (including digital pathology) data of a large cohort of Chinese breast cancer patients has contributed to the precision treatment of breast cancer.^283^

For AI algorithms, high-quality training images are required. Qualitative evaluation of AI can quickly and accurately identify cell types and provide corresponding tissue morphology and biological patterns. Integrating AI into screening and diagnostic methods, such as biopsy, can significantly improve the success rate of breast cancer screening and/or treatment. Machine learning and deep learning are the key aspects of AI in breast cancer imaging. Machine learning is used to store a large dataset, which is then used to train prediction models and interpret generalization.^284^ Deep learning is the latest branch of machine learning, which classifies and recognizes images by establishing an artificial neural network system.^285^ A prospective and population-based study found that compared with a double reading by two radiologists, replacing one radiologist with AI could induce a 4% higher non-inferior cancer detection rate.^286^ Similar findings were also observed in another randomized controlled trial.^287^ In another diagnostic accuracy cohort study, a mobile phone-AI-based infrared thermography showed significantly higher diagnostic accuracy than traditional human readers.^288^

### Multiplex immunofluorescence staining

In recent years, immunotherapy has shown promise in treating breast cancer.^289,290^ There is growing evidence that the difference in immune responsiveness is due to the heterogeneity of the tumor microenvironment.^139^ The traditional techniques for evaluating the tumor immune microenvironment, including gene expression profiling, flow cytometry, and conventional immunohistochemistry, have limitations. For example, transcriptome profiling and flow cytometry cannot obtain in situ spatial information of molecules and cells in the microenvironment. Unlike the qualitative analysis of conventional immunohistochemistry, multi-fluorescence immunohistochemistry technology has made technological innovations in multi-label staining, spectral imaging, and intelligent analysis, overcoming the limitations of traditional pathological single-label and qualitative analysis, as well as the technical shortcomings of gene expression profiling and flow cytometry that cannot obtain in situ spatial information of proteins and cells. It has obvious advantages that cannot be replaced in analyzing the tumor immune microenvironment. By using multiple fluorescence immunohistochemistry techniques, multi-channel information about cell composition and spatial arrangement can be obtained, enabling high-dimensional analysis of the tumor microenvironment and providing precise diagnosis and subsequent targeted therapy assistance for tumors.^291,292^ Clinical trials regarding multiplex immunofluorescence staining are currently limited. However, this technology has now been applied in basic experiments. For example, in a recent pilot study of patients with early-stage breast cancer who received neoadjuvant talazoparib, multiplex immunofluorescence staining was performed to examine the changes in tumor immune microenvironment.^293^ In another study, multiplex immunofluorescence imaging was also performed in combination with single-cell sequencing to depict the microenvironment of primary breast cancer.^294^ We believe that in the near future, multiplex immunofluorescence imaging will become a powerful weapon for the precision treatment of breast cancer.

## Treatment of breast cancer: emerging strategies and therapies

Advances in precise molecular subtype diagnostics have accelerated the development of systemic treatment strategies for breast cancer in recent years, particularly in the areas of endocrine therapy and anti-HER2 therapy. The continuous introduction of new drugs and clinical trials has significantly improved patient survival outcomes. As more drugs enter the neoadjuvant treatment platform, neoadjuvant therapy enhances both the precision and minimally invasive approach of local treatments. Additionally, the efficacy of the neoadjuvant platform is validated through local treatment, offering a robust foundation for adjusting the intensity and duration of subsequent adjuvant therapy.

### Local treatment

**Fig. 5:**
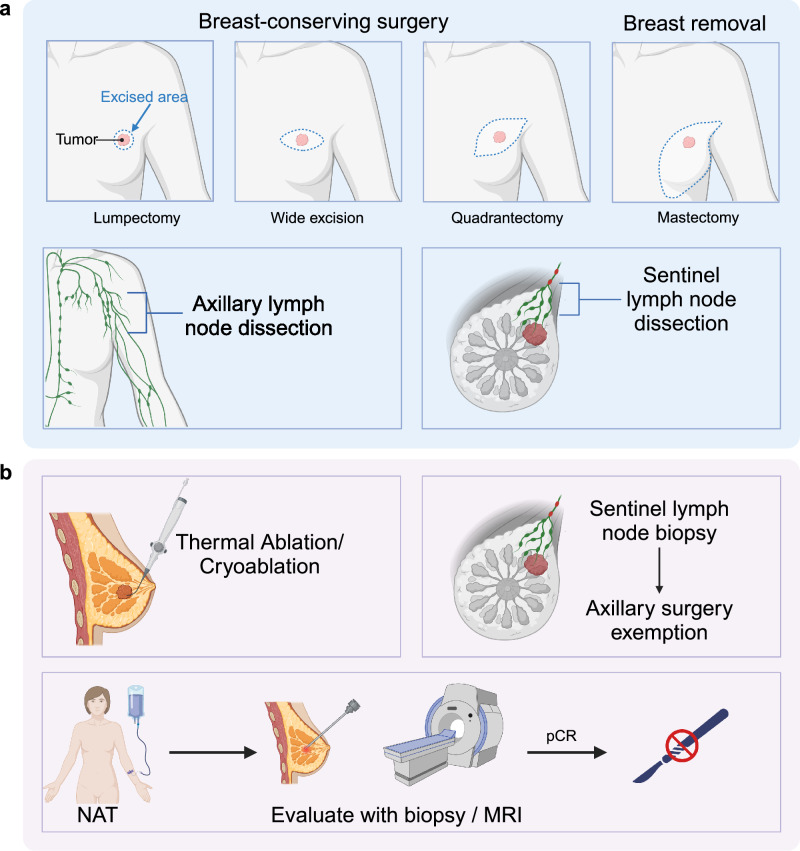
Surgical treatment for breast cancer. Traditional treatments for breast cancer include breast conservative surgeries and mastectomy, while axillary surgeries include axillary lymph node dissection and sentinel lymph node dissection. Currently, there have been some novel approaches for breast cancer treatment. Thermal ablation/cryoablation is a potential non-surgical technique for tumor destruction that can possibly replace surgical excision in some situations. Moreover, there has also been an emerging concept of using neoadjuvant therapy (NAT) to eradicate tumors and completely avoid surgery, which requires core biopsy or magnetic resonance imaging (MRI) to confirm no residual tumor. Sentinel lymph node biopsy allows safe axillary lymph node dissection exemption for certain patients. pCR pathological complete response. The figure was created with Biorender.com

Local treatment of breast cancer is undergoing revolutionary changes, with the primary goals being precise excision within the smallest possible margins and the minimization of trauma.^295^ An increasing number of patients are moving from mastectomy to breast-conserving surgery (BCS) and from axillary lymph node dissection (ALND) to sentinel lymph node biopsy (SLNB). However, this progress remains insufficient. With advancements in technology and improvements in the neoadjuvant platform, the possibility of less invasive or even surgery-free treatments is becoming a reality. Based on these principles, numerous innovative explorations are emerging in both the surgical and radiotherapy fields (Fig. 5).

#### Breast cancer ablation therapy: thermal ablation/cryoablation

Thermal ablation and cryoablation, as non-surgical techniques for tumor destruction, are increasingly being utilized for the local treatment of early breast cancer. While direct comparisons between ablation and surgical excision are limited, numerous observational studies indicate that ablation provides acceptable rates of local control and long-term survival, along with superior cosmetic outcomes.^296^ Currently, thermal ablation techniques for breast cancer include radiofrequency ablation, microwave ablation, laser ablation, and high-intensity focused ultrasound.^297–301^ Cryoablation employs extreme cold by inserting a cryoprobe into the target tissue, alternating between freeze-thaw cycles to form an ice ball that destroys the target lesion.^302^ The primary concern regarding these ablative techniques is whether they achieve efficacy comparable to breast-conserving surgery (BCS).^303^ Tumor size plays a crucial role in evaluating safety; while some studies include patients with larger tumors, more restrict inclusion to tumors no larger than 2 cm to ensure safe rates of local recurrence-free survival.^302,304–307^ Another critical factor is assessing post-ablative effectiveness, particularly the rate of complete destruction. Early studies used confirmatory surgical excision immediately after non-surgical ablations, employing dual staining methods to enhance pathological accuracy. Later studies often utilize magnetic resonance imaging and contrast-enhanced ultrasound to evaluate residual lesions intraoperatively and post-operative lesion absorption and recurrence.^308–311^

Recent research goes beyond local treatment and explores the immune response against tumors induced by ablation.^312^ During the thawing phase of cryoablation, tumor cells within the ice ball release antigens, nucleoproteins, and cytokines, recruiting macrophages and NK cells to stimulate an immune response. This leads to the release of antigen-presenting cells into the cryoablated tissue.^313–315^ Moreover, the release of tumor-specific antigens triggers specific immune responses against the tumor itself, resulting in a reduction of distant metastatic lesions known as the “abscopal effect”.^316,317^ Some centers are attempting to integrate ablation technology with immunotherapy or other targeted inhibitors to harness these treatments’ synergistic effects and safely activate the immune system, which could be pivotal in further enhancing long-term prognosis.^318,319^

#### Exploration of eliminating breast surgery

The surgical management of breast cancer is evolving towards a “less is more” approach, with a focus on preserving the Pectoralis muscle, conserving the breast tissue, and sparing the axillary lymph nodes. This advancement can be attributed mainly to conceptual innovations, particularly the introduction of neoadjuvant therapy, which provides more patients with the opportunity for surgery and breast conservation.^320^ However, even minimally invasive BCS can result in irreversible damage. Therefore, there has been an emergence of the concept of using neoadjuvant therapy to eradicate tumors and avoid surgery completely. Early clinical trials compared radiotherapy to breast surgery based on clinical complete response as the criterion for inclusion. After a follow-up period exceeding ten years, it was found that the local-regional recurrence (LRR) in the group receiving radiotherapy alone was slightly higher than those who underwent BCS followed by radiotherapy or mastectomy.^321,322^ As a result, some studies have utilized ultrasound-guided vacuum-assisted core biopsy (VACB) to obtain larger and more extensive specimens while reducing the false-negative rate to 0–5%.^323,324^ This has increased confidence in continuing standard neoadjuvant therapy for patients with cT1-2N0-1M0 in HER2+ breast cancer or TNBC. By confirming no residual tumors through multi-site VACB under ultrasound guidance in areas where imaging shows residual lesions <2 cm, these patients can avoid open breast surgery.^325^ In highly selected breast cancer patients, exemption from breast surgery is considered a potential future direction. Although some studies confirm the feasibility of exempting patients who achieve pCR after neoadjuvant systemic therapy under rigorous evaluation using ultrasound or MRI,^323,326,327^ this approach is constrained by regional development levels and technological disparities. Consequently, not all centers support this radical decision in similar studies.^328,329^

#### Exemption of axillary surgery in breast cancer

ALND has traditionally been a crucial component of breast cancer surgery. However, the theory proposed by Professor Fisher that breast cancer is a systemic disease from its onset has raised doubts about the necessity of ALND. The NSABP B-04 study revealed that 40% of patients in the radical mastectomy group had axillary lymph node metastasis, while only 18.6% of patients in the simple mastectomy group developed axillary recurrence and subsequently underwent ALND.^330^ Although the LRR rate was higher in the simple mastectomy group than in the radical surgery and mastectomy combined with axillary radiotherapy groups, there were no statistically significant differences in overall survival (OS) and distant metastasis-free survival (DMFS).^331^ This study provided a theoretical foundation for further exploration. In the NSABP B-32 study, comparing the SLNB group and SLNB + ALND group showed no significant differences in OS, disease-free survival (DFS), distant disease-free survival (DDFS), and LRR over an extended follow-up period.^332^ Subsequently, the IBCSG 23-01 study demonstrated that omitting ALND is safe and reliable when micrometastasis is found in SLNB.^333^ When considering whether to proceed with ALND after macrometastasis is detected in sentinel lymph nodes, the ACOSOG Z0011 included patients with confirmed cT1-2N0 breast cancer who underwent BCS. Regardless of whether they were followed up for 5 years or 10 years for OS, the SLNB group was found to be non-inferior to the ALND group in both follow-up periods.^334^ The AMAROS study included 18% of patients who underwent mastectomy. Although there were no statistically significant differences in DFS and OS between the radiotherapy and ALND groups over a ten-year follow-up, the primary endpoint, the 5-year axillary recurrence rate, was significantly higher in the SLNB group compared to the ALND group, failing to meet the predefined non-inferiority margin.^335^ The SENOMAC study addresses the limitations of prior research, including 36% of patients who underwent mastectomy. With OS as the primary endpoint, comparisons between the SLNB and ALND groups reveal no significant differences in breast cancer-specific survival, recurrence-free survival, or OS.^336,337^

Furthermore, the latest SOUND study employs a more pioneering approach. For patients with tumors ≤2 cm in diameter who are candidates for BCS and radiotherapy, preoperative ultrasound is used to exclude axillary lymph node metastasis. The 5-year DDFS, DFS, and OS show no statistical differences between the non-axillary surgery and SLNB groups.^338^

#### Changes in indications for radiation therapy in low-risk patients

It is widely acknowledged that adjuvant radiotherapy following BCS significantly reduces the cumulative recurrence rate in the ipsilateral breast. However, radiotherapy-related side effects are frequently encountered. Therefore, identifying low-risk patients who may be exempt from radiotherapy holds great clinical research significance.^20^ The CALGB 9343 study focuses on elderly low-risk HR+ breast cancer patients, comparing the outcomes of tamoxifen plus radiotherapy versus tamoxifen alone. The results indicate that while the radiotherapy group slightly improves LRR, this does not translate into a survival benefit.^339^ The LUMINA study extends the range to include low-risk breast cancer patients aged 55 and older who undergo only BCS and endocrine therapy. The 5-year LRR is 2.3%, with an OS of 97.2%.^340^ The PRIME 2 study enrolls patients aged ≥65 years, consistent with previous findings, the radiotherapy group demonstrates a lower ten-year LRR but no significant OS advantage.^341,342^ For elderly low-risk patients, the primary concern is how to predict the survival benefits of radiotherapy using limited information while avoiding its potential side effects.

Defining patient risk based solely on clinicopathological factors is a straightforward and practical approach, but it does have certain limitations. The IDEA (Individualized Decisions for Endocrine Therapy Alone) study represents the first application of genomic testing (Oncotype DX 21-gene) to younger postmenopausal patients who undergo endocrine therapy after BCS. This study assists in making individualized clinical decisions regarding exemption from radiotherapy and endocrine therapy alone.^343^ Other ongoing studies, such as the NRG-BR007 DEBRA trial (NCT04852887), also incorporate the Oncotype DX 21-gene recurrence score, whereas the PRECISION (NCT02653755) and EXPERT (NCT02889874) studies employ PAM50 testing. Exploring additional biomarkers will likely become a key focus in future clinical trials to accurately select patients with low risk of local recurrence and further stratify these already low-risk individuals.

### Systemic treatment for HR+/HER2− breast cancer

HR+/HER2− breast cancer represents the most common subtype and is associated with the most favorable prognosis. In recent years, as the prognostic stratification of early breast cancer has become more precise, it has become a clinical consensus to tailor the intensity and duration of endocrine therapy according to risk stratification. With advancements in molecular diagnostics, a deeper understanding of HR+/HER2− breast cancer has been achieved, and several pathway inhibitors, as well as immunotherapies, are showing increasing promise.

#### SERMs and SERDs

Tamoxifen, a selective estrogen receptor modulators (SERMs), is the classical treatment for HR+/HER2− breast cancer. Fulvestrant, an agent that acts both as an inhibitor of estrogen receptors and a selective estrogen receptor degrader (SERDs), gained approval from the U.S. Food and Drug Administration (FDA) in 2002 for the treatment of advanced hormone receptor-positive breast cancer, marking the advent of a new era in endocrine therapy.^344–346^ Although some studies indicate that breast cancer patients with ER expression between 1% and 10% may exhibit reduced sensitivity to endocrine therapy, the threshold of 1% ER expression is still used in most current clinical practices to determine the need for endocrine therapy.^347^

After the development of first-generation SERMs tamoxifen and second-generation SERMs toremifene and raloxifene, several novel endocrine therapy drugs are currently under development to enhance efficacy and reduce toxicity.^348^ While SERMs regulate ER activity instead of inhibiting it, promising results have been observed with lasofoxifene, a new-generation non-steroidal SERMs. In phase 2 studies of ELAINE-1 and ELAINE-2, lasofoxifene demonstrated significant antitumor activity in patients with *ESR1*-mutated, endocrine-resistant metastatic breast cancer.^349,350^ The ongoing phase 3 study ELAINE-3 (NCT05696626) aims to evaluate the effects of abemaciclib combined with either lasofoxifene or fulvestrant in patients with ESR1-mutated, locally advanced or metastatic ER+/HER2− breast cancer. Additionally, novel compounds such as the SERMs/SERDs hybrid bazedoxifene and the selective ER covalent antagonists are undergoing phase 2 clinical trials for patients experiencing disease progression following advanced endocrine therapy.^351^

Most SERDs with acrylic side chains are discontinued after phase 1 clinical trials due to limited efficacy and uncontrollable side effects, presenting significant challenges in developing new oral SERDs.^352^ Elacestrant has emerged as the first FDA-approved oral estrogen receptor antagonist for *ESR1*-mutant patients, owing to substantial improvements in progression-free survival (PFS) observed during the EMERALD phase 3 trial in the *ESR1*-mutant population.^353,354^ The development of the new SERDs has encountered obstacles. Amcenestrant showed potential efficacy in combination with cyclin-dependent kinases (CDK) 4/6 inhibitors for advanced HR+ breast cancer; however, both the AMEERA-3 phase 2 and AMEERA-5 phase 3 trials did not meet their efficacy endpoints for Amcenestrant as monotherapy or when combined with CDK4/6 inhibitors.^355–357^ Giredestrant, despite not reaching statistical significance for the primary endpoint of investigator-assessed PFS in the acelERA BC phase 2 study, demonstrated comparable efficacy to the physician’s choice of endocrine monotherapy in key subgroups and showed a beneficial trend in the *ESR1*-mutant population.^358^ This offers promise for further research in both the phase 2 coopERA neoadjuvant study and the phase 3 LidERA adjuvant study.^359^ Furthermore, the ongoing Phase 3 persevERA (NCT04546009) clinical trial investigates the efficacy of combining giredestrant and palbociclib as a first-line treatment for metastatic breast cancer. Camizestrant monotherapy has demonstrated reliable efficacy and safety as monotherapy for advanced HR+ breast cancer,^360^ with ongoing SERENA-4 (NCT04711252) and SERENA-6 (NCT04964934) clinical trials evaluating its efficacy when combined with CDK4/6 inhibitors. CAMBRIA-1 and CAMBRIA-2 will also evaluate the camizestrant’s efficacy as adjuvant therapy in switching settings before treatment. The ongoing clinical trials of oral SERDs primarily focus on identifying subpopulations with potential benefits following CDK4/6 inhibitor failure, investigating the efficacy of SERDs in combination with CDK4/6 inhibitors as first-line therapy, and reducing mortality rates among high-risk populations during the adjuvant stage.

### CDK4/6 inhibitors

In the field of endocrine therapy for advanced HR+ breast cancer, the combination of CDK4/6 inhibitors and aromatase inhibitors has been established as the standard treatment strategy.^361–365^ Current research is increasingly focused on early-stage treatments. Four clinical trials (PALLAS, MonarchE, NATALEE, and PENELOPE-B) have evaluated the efficacy of CDK4/6 inhibitors in adjuvant therapy.^366,367^ In earlier clinical studies, the suitability of the population for intensified treatment remains unclear, with inconsistent intensity and duration of CDK4/6 inhibitors and inadequate management of their toxic side effects. Consequently, only the Monarch E study’s experimental group demonstrates an absolute benefit of 7.6% in 5-year invasive disease-free survival (iDFS), whereas the PALLAS and PENELOPE-B trials do not achieve their primary end points.^368,369^ The NATALEE trial shows a positive outcome in iDFS.^370^ Notably, the NATALEE trial included 40% of patients who were excluded from the MonarchE study due to having stage II and stage III N0 disease, making it a more comprehensive clinical trial of CDK4/6 inhibitors in early breast cancer to date. Since patients are tumor-free during the adjuvant phase, a reduced dose regimen of 400 mg is crucial for improving patient compliance by minimizing adverse events.^371^

Integrating CDK4/6 inhibitors with traditional endocrine therapies has expanded options for neoadjuvant endocrine therapy. Studies like NeoPAL and CORALLEEN suggest that aromatase inhibitors combined with CDK4/6 inhibitors provide similar long-term survival benefits compared to neoadjuvant chemotherapy but with slightly improved breast conservation rates and fewer chemotherapy-related adverse effects.^372,373^ In neoadjuvant endocrine therapy of HR+/HER2− breast cancer, achieving pCR is not the primary goal, therefore studies such as NeoMONARCH and PALLET focus on changes in Ki67 levels and complete cell cycle arrest with the addition of CDK4/6 inhibitors.^374,375^ The FELINE study evaluates the proportion of patients achieving a post-surgical Preoperative endocrine prognostic index score of 0.^376^ Neoadjuvant endocrine therapy for HR+/HER2− early breast cancer remains less developed compared to neoadjuvant chemotherapy or targeted therapy for TNBC/HER2+ early breast cancer. The objectives, evaluation methods, regimens, duration, and subsequent intensification strategies are still being explored.

#### Novel strategies for PI3K/AKT/mTOR pathways

The activation of the phosphoinositide 3-kinase (PI3K)/protein kinase B (AKT)/mammalian target of rapamycin (mTOR) pathway plays a crucial role in endocrine resistance, positioning this pathway as a potential therapeutic target.^377^ The TAMRAD phase 2 clinical trial enrolled postmenopausal patients with HR+ metastatic breast cancer who had failed aromatase inhibitor therapy, comparing tamoxifen combined with the mTOR inhibitor everolimus versus tamoxifen monotherapy. The results demonstrate that the combination therapy group shows significantly better clinical benefit rates and PFS than the monotherapy group.^378^ Data from the BOLERO-2 phase 3 study confirm that combining endocrine therapy with everolimus offers clinical benefits for patients with HR+ advanced breast cancer who have failed non-steroidal aromatase inhibitors therapy.^379,380^ A similar conclusion is confirmed in the Phase 2 PrE0102 trial, which investigates the combination of everolimus and fulvestrant.^381^ However, the SWOG S1207 study, which aimed to add everolimus to adjuvant endocrine therapy, did not show improved efficacy for the overall population with one year of everolimus plus endocrine therapy. The completion rate of the everolimus group is lower due to side effects, and the incidence of grade 3 and 4 adverse events is higher.^382,383^ The FAKTION phase 2 trial indicates that the AKT inhibitor capivasertib combined with fulvestrant significantly improves PFS and OS in postmenopausal women with HR+/HER2− advanced breast cancer who have previously received aromatase inhibitors therapy, particularly In the AKT pathway abnormal subgroup.^384,385^ The CAPItello-291 phase 3 study indicates that regardless of AKT pathway abnormalities, the capivasertib plus fulvestrant group demonstrates a significant advantage in median PFS compared to the placebo plus fulvestrant group.^386^ This conclusion remains unchanged even when considering prior use of CDK4/6 inhibitors. Consequently, Capivasertib is approved by the FDA as the first AKT pathway inhibitor for HR+/HER2− advanced breast cancer.^387^

Regarding *PIK3CA*-mutated advanced breast cancer, the SOLAR-1 study confirms the significant PFS benefit of alpelisib combined with fulvestrant in such patients. However, CDK4/6 inhibitors were not widely used when the study was initiated.^388,389^ The BYLieve study enrolled a larger cohort of HR+/HER2− advanced breast cancer patients with *PIK3CA* mutations who had previously received CDK4/6 inhibitors along with aromatase inhibitors. These patients achieved a median PFS of 8.2 months with alpelisib plus fulvestrant or letrozole.^390^ Other clinical studies focusing on providing additional drug options for HR+ advanced breast cancer patients following CDK4/6 inhibitor use are currently ongoing within the realm of PI3K/AKT/mTOR pathway inhibitors. Table 2 provides an overview of published Phase 2 and 3 clinical trials targeting the PI3K/AKT/mTOR pathway.

**Table 2:** Phase 2/3 key clinical trials of targeted PI3K/AKT/mTOR pathways agents for patients with HR+/HER2− locally advanced or metastatic breast cancer

Target	Trial	Agent	Phase	No. of patients	Line of therapy	Arms	PFS (p)	OS (p)	References
PI3K	SANDPIPER	Taselisib	3	516	Prior ET	TAS + FUL vs. PBO + FUL	7.4 vs. 5.4 months; Hazard ratio: 0.70 (*p* = 0.0037)	NA	^547^
	SOLAR‐1	Alpelisib	3	341	Prior ET	ALP + FUL vs. PBO + FUL	11.0 vs. 5.7 months; Hazard ratio: 0.65 (*p* < 0.001)	39.3 vs. 31.4 months; Hazard ratio: 0.86 (*p* = 0.15)	^388,389^
	BYLieve	Alpelisib	2	121	Prior ET + CDK4/6i	ALP + FUL	7.3 months	17.3 months	^390^
	INAVO120	Inavolisib	3	325	Fist line	PALBO + FUL + INA vs. PALBO + FUL + PBO	15.0 vs. 7.3 months; Hazard ratio: 0.43 (*p* < 0.0001)	NA	^548^
AKT	FAKTION	Capivasertib	3	140	Prior ET	FUL + CAP vs. PBO + CAP	10.3 vs. 4.8 months; Hazard ratio: 0.58 (*p* = 0.0044)	29.3 vs. 23.4 months; Hazard ratio: 0.66 (*p* = 0.035)	^384,385^
	CAPItello-291	Capivasertib	3	708	Prior ET ± CDK4/6i	CAP + FUL vs. PBO + FUL	7.2 vs. 3.6 months; Hazard ratio: 0.60 (*p* <0.001)	NA	^386^
	IPATunity130, cohort B	Ipatasertib	3	222	No prior CT for ABC or relapse >1 year of NAC	IPAT + PAC vs. PBO + PAC	9.3 vs. 9.3 months; Hazard ratio: 1.00	NA	^549^
mTOR	TAMRAD	Everolimus	2	111	Prior ET	EVE + TAM vs. TAM	8.6 vs. 4.5 months; Hazard ratio: 0.54	NA	^378^
	BOLERO‐2	Everolimus	3	724	Prior ET	EVE + EXE vs. PBO + EXE	7.8 vs. 3.2 months; Hazard ratio: 0.45 (*p* < 0.0001)	31.0 vs. 26.6 months; Hazard ratio: 0.89 (*p* = 0.14)	^380,550^
	PrE0102	Everolimus	2	131	Prior ET	FUL + EVE vs. FUL+ PBO	10.3 vs. 5.1 months; Hazard ratio: 0.61 (*p* = 0.02)	NA	^381^

*HR* hormone receptor, *HER2* human epidermal growth factor receptor-2, *ET* endocrine therapy, *TAS* taselisib, *FUL* fulvestrant, *PBO* placebo, *ALP* alpelisib, *CAP* capivasertib, *CT* chemotherapy, *ABC* advanced breast cancer, *NAC* neoadjuvant chemotherapy, *IPAT* patasertib, *PAC* paclitaxel, *EVE* everolimus, *TAM* tamoxifen, *EXE* exemestane, NA not available, PALBO palbociclib, INA inavolisib

#### Immune checkpoint inhibitor treatment

HR+/HER2− breast cancer, the most prevalent subtype, exhibits limited sensitivity to neoadjuvant chemotherapy, with a pCR rate of ~10%.^391^ While endocrine therapy remains pivotal in the treatment of HR+/HER2− breast cancer, previous research indicates that neoadjuvant endocrine therapy does not significantly enhance the pCR rate.^392^ Immune checkpoint inhibitors (ICIs) therapy has demonstrated remarkable effectiveness in TNBC, prompting numerous centers to investigate its perioperative value in HR+/HER2− breast cancer. In the I-SPY2 trial, the cohort with HR+/HER2− tumors revealed that combining neoadjuvant pembrolizumab with chemotherapy effectively increases the pCR rate.^289^ Another cohort demonstrated that combining durvalumab, olaparib, and chemotherapy not only elevates the pCR rate in HR+/HER2− patients but also identifies ultra-high-risk populations who benefit more from this combined treatment through MammaPrint gene testing.^290^ Phase 3 clinical trials CheckMate 7FL and KEYNOTE-756 have reported improved pCR rates with neoadjuvant chemotherapy and ICI treatment. In the CheckMate 7FL trial, patients with PD-L1 IC (Immune cell) ≥1% show greater sensitivity to immunotherapy. However, in the KEYNOTE-756 trial, patients with low-ER expression (<10%) exhibit a more significant increase in pCR. These findings suggest that the sensitive populations for immunotherapy in the neoadjuvant setting may differ between HR+ and TNBC patients.^393,394^ The subsequent survival data from these trials will further strengthen our confidence in pursuing additional immunotherapy trials among high-risk and favorable populations. However, not all attempts at immunotherapy prove satisfactory. In the case of metastatic breast cancer, combining ipilimumab and nivolumab with anthracycline-based chemotherapy leads to an escalation in toxic side effects without yielding any clinical benefits.^395^ Identifying the population that may truly benefit from ICI therapy remains a critical issue to address in the foreseeable future.

### Systemic treatment for HER2+ breast cancer

The prognosis of HER2+ breast cancer has significantly improved due to the development and refinement of anti-HER2 therapies. Key considerations in this area include the potential for reducing or omitting chemotherapy in low-risk patients and optimizing the combination and prioritization of different anti-HER2 drugs for high-risk patients.

#### Chemotherapy exemption

Chemotherapy de-escalation has now gained more attention as a potential standard of care for breast cancer.^396^ As for the early stage of HER2+ breast cancer, the combination of trastuzumab and pertuzumab with chemotherapy has emerged as the standard therapeutic approach. In recent years, the question of whether there is a role for de-escalation of systemic neoadjuvant and adjuvant therapies has arisen. Numerous attempts have been made to de-escalate medical therapy in selected patients. The Adjuvant Paclitaxel and Trastuzumab (APT) trial first explored some patients with low-risk HER2+ breast cancer who may not require adjuvant trastuzumab-based chemotherapy.^397,398^ The KAITLIN phase 3 study compared anthracycline-based chemotherapy and then 18 cycles of T-DM1 plus pertuzumab (AC-KP) to taxane (three–four cycles) plus trastuzumab plus pertuzumab (AC-THP), adjuvant AC-KP did not result in statistically significant or clinically meaningful improvement in iDFS compared with AC-THP in patients with high-risk early breast cancer.^399^

In HR+/HER2+ breast cancer, there is potential for endocrine therapy to replace chemotherapy in combination with anti-HER2 drugs. In the SYSUCC-002 study, endocrine therapy combined with trastuzumab demonstrates efficacy not inferior to that of chemotherapy combined with trastuzumab, while also reducing the toxic side effects associated with chemotherapy and improving patient compliance.^400^ The MonarcHER study similarly indicates the potential for combining CDK4/6 inhibitors with fulvestrant as a companion to trastuzumab.^401,402^ Further clinical trials are underway to explore combinations of various endocrine therapies with anti-HER2 regimens. In the WSG-ADAPT-HER2+/HR- trial, a subgroup of patients with HER2+ early breast cancer exhibited promising efficacy to anti-HER2 treatment alone without chemotherapy; more than a third of the patients achieved a pathological complete response with a chemotherapy-free regimen just containing dual HER2 blockade treatment.^403,404^ The PHERGain study explored whether it is possible to treat patients with early HER2+ breast cancer with dual antibody therapy alone in both neoadjuvant and adjuvant settings, completely sparing chemotherapy. This study showed that a subgroup of patients using this innovative de-escalating approach omitting chemotherapy received a pCR response, and around one-third of the patients achieved a promising 3-yeat iDFS from surgery with chemotherapy-free therapy.^405,406^

Given the favorable outcomes reported in contemporary studies of HER2+ breast cancer, ongoing researches are now focused on strategies to de-escalate chemotherapy while maintaining optimal HER2−targeted therapy with trastuzumab plus pertuzumab in the majority of patients.^407^ Selecting potential subgroups for chemotherapy de-escalation still needs further validation.

#### Anti-HER2 ADCs

Antibody-drug conjugates (ADCs) are a rapidly developing therapeutic approach in cancer treatment that has shown remarkable activity in breast cancer. ADCs contain three major parts: a monoclonal antibody, a chemical linker, and cytotoxic payloads.^408^ Upon being given intravenously, the circulating ADCs bind to tumor targets and initiate endocytosis. The endocytosed endosomes then fuse with lysosomes, where ADCs undergo lysosomal degradation and freeing of cytotoxic payloads into the cytosol.^409^

Trastuzumab emtansine (T-DM1) is the first approved ADC for advanced HER2+ breast cancer.^410,411^ Remarkably, patients with HER2+ advanced breast cancer achieved a significantly longer PFS using trastuzumab deruxtecan (T-DXd) than T-DM1 in the DESTINY-Breast03 trial.^412^ Notably, the DESTINY-Breast series of studies has demonstrated significant benefits for patients with brain metastases, leading to T-DXd replacing TDM-1 and TKIs as the standard second-line treatment for advanced HER2+ breast cancer. The ongoing DESTINY-Breast09 study aims to challenge the position of taxane combined with dual-targeted therapy, potentially advancing T-DXd to the first-line treatment stage for advanced anti-HER2 therapy. In the adjuvant setting, the KATHERINE study offers non-pCR patients after neoadjuvant therapy an opportunity to switch to alternative anti-HER2 drugs, marking a satisfactory exploration.^413^ Similarly, SHR-A1811 and T-DXd are also undergoing similar trials. In the neoadjuvant settings, the KRISTINE trial compared T-DM1 plus pertuzumab to the standard of care in early HER2+ breast cancer. However, T-DM1 failed to show superior clinical outcomes compared to the control group.^414,415^ A comparable result was also observed in a phase 2 trial (NCT02568839), which compared neoadjuvant T-DM1 monotherapy to the current standard treatment in HER2+ breast cancer. Similar pCR rates were reached in both arms.^416^ Furthermore, in DESTINY-Breast11 neoadjuvant T-DXd is being tested as the single regimen for early HER2+ breast cancer. These results are still awaiting and possibly change the future of the treatment for HER2+ breast cancer. Of note, T-DXd has been explored in HER2−low breast cancer (either ER+ or ER−) and as an alternative option for these patients with advanced disease.^417^

#### HER2 vaccine

Cancer vaccines are an immune-based treatment strategy that boosts an antitumor immune response by activating a wide range of immune regulators such as cytotoxic lymphocytes, antibodies, and Th cells in the patient to induce a therapeutic effect.^418,419^ Immunogenic HER2−derived peptides include peptides from different parts of the HER2 molecule consisting of E75 (from the extracellular domain), AE37 (ICD), and GP2 (transmembrane domain). The E75 (nelipepimut-S) vaccine showed promising efficacy in stimulating an in vivo immune response and might reduce the disease recurrence rate in disease-free, node-positive, and high-risk node-negative breast cancer patients.^420^ Based on these data, the E75+ granulocyte-macrophage colony-stimulating factor, now known as NeuVax, is being evaluated in a phase 3 trial. A plasmid DNA vaccine encoding the HER2 ICD showed safety and efficacy in patients with advanced HER2+ breast cancer.^421^ This HER2 ICD vaccine is now being evaluated as adjuvant treatment in an ongoing randomized clinical trial.

#### Strategies for HER2 heterogeneity

HER2+ breast cancer is a biologically heterogeneous disease. HER2 heterogeneity has been reported in up to 40% of breast cancers.^422^ Some studies also showed that HER2 heterogeneity correlated with worse clinical outcomes,^423^ and HER2 intra-tumoral heterogeneity could be another potential factor for the resistance to anti-HER2 therapy. Given the relatively poor response to anti-HER2-targeted therapy in breast cancer patients with HER2 heterogeneity, the treatment regimens can be deescalated or escalated according to the HER2 status. According to a recent retrospective clinical study, patients with various statuses of HER2 (heterogenous, reduced, and loss) benefited from T-DXd,^424^ while these patients showed limited response to T-DM1.^425,426^

### Systemic treatment for TNBC

TNBC represents a highly heterogeneous group of tumors, and the therapeutic efficacy of traditional chemotherapy appears to have reached a plateau. With advancements in molecular diagnostics, more precise subtyping of triple-negative breast cancer has been achieved, leading to the introduction of new treatment strategies that, when combined with chemotherapy, have significantly improved patient outcomes.

#### Targeting BRCAness

Pathogenic mutations of *BRCA1*/*2* are common in TNBC. *BRCA1*/2-mutations typically cause homologous recombination deficiency (HRD), which provides susceptibility to DNA crosslink agents or poly (ADP-ribose) polymerase (PARP) inhibitors. The platinum agent introduces intra-strand crosslinks, which eventually lead to cell apoptosis in the absence of proper functioning of *BRCA1/2*. Meta-analysis showed that platinum-based regimens significantly improved objective response rate (ORR), pCR, DFS, and OS compared with patients without platinum-based chemotherapy regimens.^427^ The TNT study reported that in *BRCA*-mutated patients, ORR and median PFS were significantly higher in the carboplatin group than in the docetaxel group.^428^ A meta-analysis evaluating platinum-based vs. platinum-free neoadjuvant chemotherapy found that the use of platinum neoadjuvant chemotherapy significantly improved the pCR rate in TNBC patients.^429^ This meta-analysis also explored the relationship between *BRCA* gene mutation status and the efficacy of platinum drugs; however, no significant relationship was found.^429^ Platinum-based drugs have become an indispensable element in the neoadjuvant treatment phase following anthracyclines and taxanes. However, their role in the adjuvant treatment phase remains controversial. The PATTERN study first confirmed the benefit of carboplatin combined with paclitaxel over the cyclophosphamide, epirubicin, fluorouracil, and docetaxel regimen in the adjuvant settings in TNBC patients; however, it failed to confirm the benefit of platinum-based adjuvant chemotherapy for patients with *BRCA* gene mutation.^430^

The PARP cluster of polymerase enzymes controls genetic stability and DNA repair. Inhibition of PARP contributes to *BRCA*-mutated tumor cell death due to synthetic lethality. After the first developed PARP inhibitor, olaparib, multiple PARP inhibitors such as niraparib, veliparib, and talazoparib were subsequently developed and tested in different phases of clinical trials. By now, olaparib and talazoparib have received FDA and European Medicines Agency approval for the treatment of patients with germline *BRCA*-mutated HER2− metastatic breast cancer based on the results of the phase 3 OlympiAD and EMBRACA trials, respectively.^431,432^ Patients receiving PARP inhibitors had a significantly longer median PFS and a higher ORR than those receiving treatment of the physician’s choice.

As for early breast cancer, Combining PARP inhibitors and standard chemotherapeutic agents in the treatment of cancer could be challenging due to toxicities. The phase 2 GeparOLA study compared paclitaxel + olaparib and paclitaxel + carboplatin in neoadjuvant settings in patients with HER2−, HRD+ early breast cancer. No statistically significant pCR differences were noticed between the groups, however, the subgroup analysis showed that for HR+ patients under 40 years old, combination therapy with paclitaxel + olaparib can lead to higher pCR rates.^433^ In the BrighTNess study, which evaluated veliparib in neoadjuvant settings in early-stage TNBC patients, showed that the addition of veliparib neither improved the rate of pCR vs standard chemotherapy nor the event-free survival.^434^ However, in another study targeting germline *BRCA1/2* wild-type TNBC, it is found that neoadjuvant olaparib does not improve pCR rates, event-free survivial (EFS), or OS when added to carboplatin-paclitaxel and anthracycline-based chemotherapy.^435^ We look forward to subsequent translational studies to identify specific baseline or post-treatment biomarkers that may more accurately predict subgroups sensitive to olaparib therapy. PARP inhibitors have also been studied in adjuvant settings. In the OlympiA study, one year of olaparib adjuvant therapy after completion of local therapy and (neo)adjuvant chemotherapy significantly improved the 3-year iDFS and DDFS compared to placebo.^436^

Many other trials are also exploring the use of PARP inhibitors in TNBC patients with *BRCA1/2* mutation, which is expected to further enrich the comprehensive treatment landscape of TNBC at different stages.

#### Anti-Trop2 ADCs

Trophoblast cell surface antigen 2 (Trop2) is a transmembrane glycoprotein that serves as a transducer for intracellular calcium signaling. It appears to be a preferable target due to its overexpression in 80% of TNBC.^437,438^ Sacituzumab govitecan (SG) received its first FDA approval for treating metastatic TNBC patients according to the results of the ASCENT trial.^439^ This trial investigated the efficacy of SG in advanced TNBC patients with at least two prior treatments, and SG significantly improved PFS and OS compared with physician’s choices.^439^ The absence of target expression has been noticed in a TNBC patient with de novo resistance to SG. In the same study, mutated *TACSTD2* (encoding Trop2) was detected in another patient with acquired resistance to SG, which led to reduced antibody binding due to an abnormal subcellular localization of Trop2.^440^ Surprisingly, although the appearance of Trop2 is necessary, a higher level of Trop2 does not necessarily result in a better response.^441^

Beyond SG, other anti-Trop2 ADCs are also emerging in our field of view. The OptiTROP-Breast01 study demonstrates that sacituzumab tirumotecan monotherapy shows benefits in PFS and OS compared to chemotherapy in advanced TNBC.^442^ Similarly, the TROPION-Breast01 study shows positive results with Dato-DXd.^443^ Researchers took more investigations beyond monotherapy. The BEGONIA study is currently evaluating the combination of durvalumab with other novel therapies as a first-line treatment for advanced TNBC. Recent results indicate that Dato-DXd combined with durvalumab demonstrates impressive efficacy and manageable safety and tolerability.^444^ Ongoing TROPION-Breast02 and -Breast05 studies aim to further explore the efficacy of Dato-DXd monotherapy and its combination with other immunotherapy regimens as first-line treatment for TNBC. Additionally, TROPION-Breast03 and -Breast04 studies are evaluating the efficacy and safety of Dato-DXd as adjuvant monotherapy and in combination with neoadjuvant immunotherapy, respectively.

#### Immunotherapy

The emergence of cancer immunotherapy has brought about revolutionary advancements in the field of cancer treatment.^445^ Immunotherapeutic treatments have shown significant efficacy in TNBCs due to tumor immune infiltration, neoantigens caused by mutational burden and higher genomic instability, and high levels of immune markers such as PD-L1 and programmed cell death 1 (PD-1).^446^ Targeting the PD-1/PD-L1 axis has become the research focus for TNBC immunotherapy. In the KEYNOTE-119 trial, monotherapeutic pembrolizumab did not significantly improve OS in patients with previously treated metastatic TNBC compared to chemotherapy, which might inform future research of pembrolizumab monotherapy for selected patients, specifically those with PD-L1-enriched tumors, and combinatorial approach might be necessary for the treatment of patients with metastatic TNBC.^447^ In multiple trials, ICIs and chemotherapy could exert synergistic effects. In the KEYNOTE-355 trial, pembrolizumab combined with chemotherapy significantly extends OS in patients with metastatic TNBC with a CPS ≥ 10, making it the first immunotherapy to achieve this. Among different chemotherapy regimens, only those patients treated with taxane (including steroid premedication) in combination with pembrolizumab show improved OS. In contrast, those treated with nab-paclitaxel monotherapy or gemcitabine combined with carboplatin do not exhibit increased OS when pembrolizumab is added.^448,449^ IMpassion130 also showed that atezolizumab plus nab-paclitaxel administered as first-line treatment produced significantly longer PFS in both the intention-to-treat population and the subgroup of patients with PD-L1-positive tumors.^450^ However, in the phase 3 IMpassion131, the combination of atezolizumab with paclitaxel did not improve PFS or OS versus paclitaxel alone in patients with locally advanced TNBC,^451^ making the role of atezolizumab in advanced disease controversial and needed to be further confirmed.

Among patients with early TNBC, as in the KEYNOTE-522 trial, neoadjuvant pembrolizumab plus chemotherapy, followed by adjuvant pembrolizumab after surgery, resulted in significantly higher pCR, especially longer EFS and os than neoadjuvant chemotherapy alone.^452,453^ Besides, in the IMpassion031 study, neoadjuvant treatment with atezolizumab in combination with nab-paclitaxel and anthracycline-based chemotherapy significantly improved pCR rate in patients with early TNBC.^454^ However, the IMpassion030 trial failed to demonstrate the efficacy of adjuvant atezolizumab in combination with chemotherapy.^455^ Currently, the standard immunotherapeutic agent for treating TNBC is pembrolizumab, and more exploration is required to establish the efficacy of immunotherapy as well as the predictive biomarker in TNBC.

CTLA-4 inhibitory signal pathway between antigen-presenting cells and lymphocytes prevents lymphocytes from being overactivated.^456,457^ Anti-CTLA-4 antibodies are confined to trigger intracellular signal pathways, including cell cycle regulation pathways which do not overlap with anti-PD-1/PD-L1 antibodies.^458^ CTLA-4 inhibitor ipilimumab has been approved by the FDA for the treatment of advanced metastatic melanoma, while it also exhibited modest antitumor activity observed in TNBC.^459^ In first-line treatment of metastatic TNBC, the anti-PD-L1/CTLA-4 bispecific antibody KN046, combined with nab-paclitaxel, shows significant efficacy and survival benefits.^460^ A phase 2 clinical trial evaluating the combination of anti-PD-L1 antibody durvalumab, anti-CTLA-4 antibody tremelimumab, and PARP inhibitors in multiple solid tumors is ongoing.^461^ Moreover, decreasing tumor competition for glucose may facilitate the therapeutic activity of CTLA-4 blockade, thus supporting its combination with inhibitors of tumor glycolysis.^462^

Other immunotherapies for TNBC include cancer vaccines and chimeric antigen receptor-modified T cells (CAR-T). The neoantigen with strong immunogenicity is derived from tumor mutant protein. Neoantigen takes full account of individual differences, and cancer vaccines targeting neoantigens could improve the inhibitory activity of immune surveillance sites.^463^ In a randomized phase 2 trial compared paclitaxel + durvalumab + neoantigen vaccine to paclitaxel + durvalumab reported, the clinical efficacy of neoantigen vaccines in TNBC is currently being evaluated.^464^ CAR-T therapy is another novel immunotherapeutic attempt for TNBC. A phase 1 trial included 4 *ROR1*-positive TNBC patients who had undergone at least three-line treatments and received *ROR1*-targeted CAR-T therapy, 2 individuals showed stable disease, and one participant had a partial response after the second infusion, persisting for 14 weeks.^465^ From a more novel perspective, the findings that intralesional oncolytic herpes virus treatment enhances anticancer immune responses in immunosuppressive tumor microenvironments provide a biological rationale for the use of this oncolytic modality in cancers that are otherwise unresponsive to immunotherapy.^466^

The future treatment landscape of TNBC will probably involve novel combinations to extend the population of patients who might benefit from immunotherapies. The potential companion drugs should synergize with ICIs or support the immune-modulating properties. The combination of ICIs with PARP inhibitors and ADCs has shown promising results.^467,468^ Table 3 presents notable clinical studies regarding the application of ICIs in the treatment of TNBC.

**Table 3:** Phase 2/3 key clinical trials of ICIs involving patients with TNBC

Line of therapy	Trial	Agent	Phase	No. of patients	Arms	Primary endpoint	Secondary endpoint	References
First line of ABC	IMpassion130	Atezolizumab	3	902	Nab-P + ATE vs. PBO + ATE	PFS:7.5 vs. 5.0 months; Hazard ratio: 0.62 (*p* < 0.0001)	OS:25.4 vs. 17.9 months; Hazard ratio: 0.67	^450,551^
	IMpassion131	Atezolizumab	3	651	PAC + ATE vs. PBO + ATE	PFS:6.0 vs. 5.7 months; Hazard ratio: 0.82 (*p* = 0.2)	OS:22.1 vs. 28.3 months; Hazard ratio: 1.11	^451^
	KEYNOTE-355	Pembrolizumab	3	847	CT + PEMBRO vs. CT + PBO	PFS:9.7 vs. 5.6 months; Hazard ratio: 0.65 (*p* = 0.001)	OS:23.0 vs. 16.1 months; Hazard ratio: 0.73 (*p* = 0.0185)	^448,449^
	TORCHLIGHT	Toripalimab	3	531	Nab-P+toripalimab vs. PBO+ Nab-P	PFS:8.4 vs. 5.6 months; Hazard ratio: 0.65 (*p* = 0.0102)	OS:32.8 vs. 19.5 months; Hazard ratio: 0.62 (*p* = 0.0148)	^552^
NST	I-SPY2	Pembrolizumab	2	114	PAC ± PEMBRO	pCR: 60% vs. 22%		^289^
	GeparNUEVO	Durvalumab	2	174	nab-P+durvalumab vs. nab-P + PBO	pCR: 53.4% vs. 44.2% (*p* = 0.287)	iDFS: 85.6% vs. 77.2%; Hazard ratio: 0.48 (*p* = 0.036)	^553,554^
	KEYNOTE-522	Pembrolizumab	3	1174	CT + PEMBRO vs. PBO + PEMBRO	pCR: 64.8% vs. 51.2% (*p* < 0 .001)	The 36-month EFS rate: 91.2% vs 77.2%	^452,555,556^
	IMpassion031	Atezolizumab	3	313	nab-P + ATE vs. nab-P + PBO	pCR: 58% vs. 41% (*p* = 0.004)	EFS: Hazard ratio: 0.76	^454^
	NeoTRIPaPDL1	Atezolizumab	3	280	nab-P+ Cb ±ATE	pCR:48.6% vs. 44.4% (*p* = 0.48)	EFS: NA	^557^
AST	IMpassion030	Atezolizumab	3	2199	CT + ATE vs. CT	iDFS: Hazard Ratio: 1.1		^558^

*ICIs* immune checkpoint inhibitors, *PBO* placebo, *CT* chemotherapy, *ABC* advanced breast cancer, *NST* neoadjuvant systemic treatment, *PAC* paclitaxel, *Cb* carboplatin, *Nab-P* Nab-paclitaxel, *ATE* atezolizumab, *PEMBRO* pembrolizumab, *AST* adjuvant systemic treatment, *EFS* event-free survival

#### Other novel regimens

There are multiple molecular targeted therapies for TNBC aside from those mentioned above. Androgen receptor (AR) targeted therapy is particularly effective in a subset of patients with TNBC that express AR. In a phase 2 study, AR inhibitor enzalutamide demonstrated clinical activity and was well tolerated in patients with advanced AR-positive TNBC.^469^ In another phase 2 trial, the combination of AR inhibitor and ICI showed a modest clinical benefit rate of 25% at 16 weeks in heavily pretreated AR-positive TNBC without preselected PD-L1.^470^

The PI3K/AKT/mTOR signaling pathway is frequently activated in TNBC. In the phase 1/2 trial, alpelisib (a PI3K inhibitor) plus nab-paclitaxel was well tolerated and showed encouraging efficacy, especially in patients with metastatic *PIK3CA*-mutated HER2− breast cancer, despite that TNBC has lower efficacy than HR+/HER2− patients.^471^ According to the PAKT trial, the addition of the AKT inhibitor capivasertib to the first-line paclitaxel therapy for TNBC resulted in significantly longer PFS and OS, especially in tumors with PIK3CA/AKT1/PTEN alterations.^472^ Besides, ipatasertib was also tested in combination with neoadjuvant chemotherapy in the phase 2 FAIRLANE study, where this regimen showed numerically higher ORR compared to placebo.^473^ Novel agents targeting the PI3K/AKT/mTOR signaling pathway have shown a certain but limited degree of efficacy in TNBC, and routine application is not recommended. Future efforts should focus on identifying potentially effective patient subgroups.

Vascular endothelial growth factor (VEGF) and its tyrosine kinase receptor VEGFR support the growth and maintenance of tumor neovasculature necessary for survival and metastasis. Bevacizumab is an anti-angiogenic agent that specifically targets VEGF.^474^ The addition of bevacizumab to chemotherapy benefits patients with metastatic breast cancer and those with stage II-III TNBC in the neoadjuvant settings.^475,476^ In the phase 3 GeparQuinto trial, bevacizumab was added to patients treated with anthracycline and taxane, which significantly improved the pCR rate in *BRCA1*/2 mutation carriers.^477^ Aside from the anti-VEGF monoclonal antibody bevacizumab, VEGFR inhibitors such as apatinib and lenvatinib can also block VEGFR2 signaling. The combination of apatinib and ICIs demonstrated favorable therapeutic effects and a manageable safety profile in patients with advanced TNBC.^478^ In contrast, the final OS analysis of the phase 3 BEATRICE trial showed no significant benefit from bevacizumab therapy for early TNBC.^479^ Therefore, the clinical utility of anti-angiogenic agents in the management of TNBC varies between early-stage and advanced-stage disease.

**Fig. 6:**
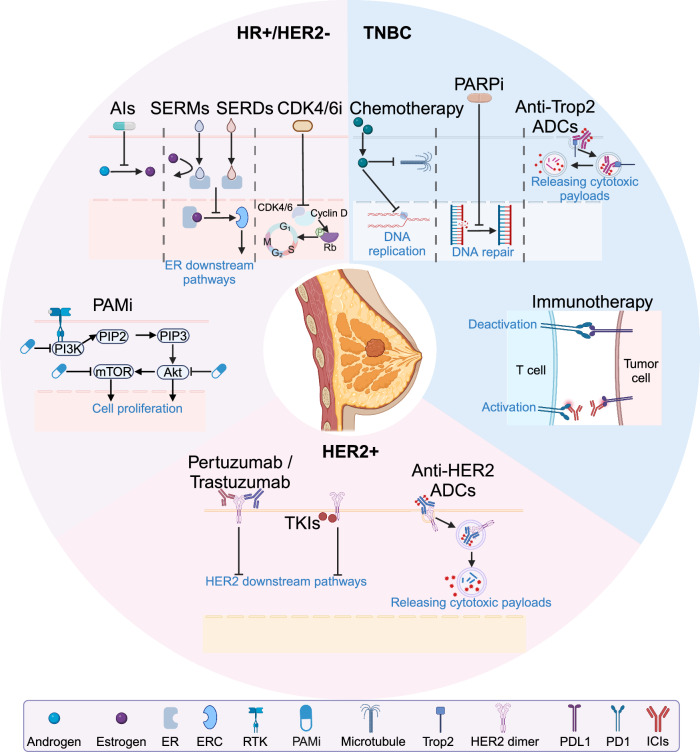
Systematic treatment for breast cancer. In HR+/HER2− breast cancer, aromatase inhibitors (AIs) are a traditional regimen for estrogen synthesis inhibition. Selective estrogen receptor modulators (SERMs) and selective estrogen receptor degraders (SERDs) started the new era of endocrine therapy for HR+/HER2− breast cancer. Cyclin-dependent kinases (CDK) 4/6 inhibitors are another novel treatment strategy for HR+/HER2− breast cancer. Furthermore, the inhibition of the phosphoinositide 3-kinase (PI3K)/protein kinase B (AKT)/mammalian target of rapamycin (mTOR) pathway also showed promising clinical efficacy. In HER2+ breast cancer, dual HER2−targeted therapy with trastuzumab and pertuzumab is established as the standard treatment. Following the failure of conventional anti-HER2 therapies, the introduction of tyrosine kinase inhibitors (TKIs) and antibody-drug conjugates (ADCs) continues to extend patients’ clinical prognosis through distinct mechanisms of action. For triple-negative breast cancer (TNBC), chemotherapy has long been a classic and effective treatment approach. In recent years, advances in translational medicine have expanded treatment options with the introduction of Poly (ADP-ribose) polymerase inhibitors (PARPi) and anti-Trophoblast cell surface antigen 2 (Trop2) ADCs. Notably, in the era of immunotherapy, immune checkpoint inhibitors (ICIs) have shown promising results in both advanced cases and in early-stage neoadjuvant settings. HR hormone receptor, HER2 human epidermal growth factor receptor-2, ER estrogen receptor, ERC ER coactivator, Rb retinoblastoma-associated protein, PAMi PI3K/AKT/mTOR pathway inhibitors, RTK receptor tyrosine kinase, PD-1 programmed cell death 1, PD-L1 programmed cell death 1 ligand 1. The figure was created with Biorender.com

In summary, advancement in understanding the tumorigenesis and progression of breast cancer form the foundation for improvements in systemic treatment. The paradigm of systemic therapy has shifted from traditional methods to subtype-specific and personalized approaches (Fig. 6). The primary objective in our future endeavors is to meticulously determine the optimal medication for each individual at the most opportune moment.

## Quality of life and long-term management of patients

Tumor is considered a systemic disease. Beyond the malignancy itself, breast cancer patients experience challenges such as treatment side effects and psychological issues, which significantly affect their quality of life. Therefore, long-term management strategies for breast cancer patients must be explored to enhance their overall well-being.

### Comprehensive rehabilitation plan

For breast cancer patients, chemotherapy toxicity and ovarian dysfunction are major challenges that significantly impact the quality of life for some individuals.

#### Reducing chemotherapy toxicity

Toxicity induced by chemotherapy in breast cancer included fatigue, insomnia, nausea and vomiting, arrest of bone marrow, alopecia, peripheral neuropathy, cognitive impairment, cardiotoxicity, and liver and kidney dysfunction, etc. Patients with fatigue and insomnia could receive moderate-intensity exercise and/or cognitive-behavioral therapy.^480–483^ For patients with nausea and vomiting, dietary therapy can be used, and greasy and/or spicy foods should be avoided in the meantime. When vomiting is severe, maintaining electrolyte balance should be paid attention to. Other methods, such as auricular acupressure, can also be attempted.^484^ Insomnia patients could receive hypnotics such as zolpidem or melatonin.^482,483,485^ Chemotherapy-induced alopecia often has an emotional impact on patients and may even lead to refusal of treatment. For this issue, the cold hat or scalp cooling system that has emerged in recent years has shown promise and is a good choice for these patients.^486,487^ In the SCALP randomized clinical trial, effective hair preservation was achieved in 50.5% of patients who received scalp cooling versus none of the patients in the control group.^488^ In another prospective multi-center cohort study, less than 50% of hair loss was defined as treatment success, and the scalp cooling group was found to induce a significantly higher success rate compared with the control group.^489^ As for the treatment time, there is evidence that for patients with luminal A breast cancer, delayed chemotherapy would not increase the recurrence risk, while for patients with other subtypes of breast cancer, every four weeks of delay would significantly affect the prognosis of patients.^490^

Peripheral neuropathy can be treated by acupuncture, functional and balance training.^491,492^ Patients with cognitive impairment can receive pharmacological donepezil or modafinil treatment or relaxation and mindfulness training.^493–498^ Cardiotoxicity can be treated by corresponding cardiovascular drugs. For patients with liver and kidney dysfunction, firstly, drugs that can worsen liver and kidney dysfunction should be avoided. Patients with liver dysfunction should receive corresponding liver protection treatment. Patients with severe renal dysfunction can receive dialysis treatment or even kidney transplantation treatment.

#### Fertility preservation

Chemotherapy may also damage the reproductive system of breast cancer patients and may cause abnormal menstrual cycle and even infertility. For this issue, other than traditional pharmacological ovarian suppression such as goserelin,^499^ patients can also receive a diet that is beneficial to their reproductive organs while avoiding behaviors that are harmful to reproductive health, such as smoking and drinking. The application of ovarian suppression in breast cancer treatment has been well-studied in clinical trials. The early meta-analysis indicated that gonadotropin-releasing hormone agonists can well protect the ovarian function of breast cancer patients during chemotherapy.^500^ A more recent meta-analysis, including 7 larger randomized trials, also indicated that gonadotropin-releasing hormone agonists were associated with increased recovery rates of regular menses.^501^ Similarly, a phase 3, randomized controlled trial (NCT01712893) also proved the ovarian preservation function of gonadotropin-releasing hormone agonists in breast cancer treatment during chemotherapy.^502^ As for the risk of recurrence among women who attempted to conceive, a recent prospective trial indicated that among HR+ early breast cancer patients, temporary interruption of endocrine therapy to attempt pregnancy would not induce a higher rate of breast cancer recurrence (8.9%) compared to the control group (9.2%).^503^ Additionally, optimization of chemotherapy regimens may also reduce the incidence of premature ovarian failure and increase the likelihood of successful pregnancies. In a recent phase 3 study, the cyclophosphamide-free adjuvant chemotherapy (epirubicin and paclitaxel followed by weekly paclitaxel) was associated with a higher probability of menses resumption, compared to the cyclophosphamide-containing regimen (epirubicin and paclitaxel followed by weekly paclitaxel).^504^

If patients have reproductive needs, they can consult a doctor for more personalized advice and choose reproductive methods that are safe and appropriate to them. Patients can also use drugs that protect the ovaries or even freeze eggs to ensure their fertility. For male patients with breast cancer, chemotherapy may induce a decline in the number and quality of sperm. Therefore, they can consider freezing sperm before treatment to ensure their fertility. The onset age of hereditary breast cancer is usually low. For this part of the population, it is more important to protect fertility. Data from a retrospective study indicated that hereditary breast cancer patients are associated with significantly higher numbers of cryopreserved embryos than non-hereditary breast cancer patients.^505^

### Digital health and telerehabilitation

Telerehabilitation in breast cancer patient management is relatively nascent, but it is expected to reduce disease burden and functional damage caused by surgery and pharmacological toxicity. It thus may have a more significant impact on the rehabilitation of breast cancer patients. The outcome measurement of telerehabilitation for cancer treatment includes functional motor ability, anxiety score, and quality of life. Telerehabilitation technology can also provide a broader evaluation of cancer care effectiveness through digital examinations.^506,507^ In addition, telerehabilitation can also be applied in palliative care, which can reduce symptoms, improve patients’ comfort, and increase the satisfaction of families.^508^

Chatbots dedicated to imitating human language are playing an increasingly important role in clinical management with the development and maturity of AI technology. AI companies, large electronic healthcare systems, and companies with healthcare systems are all striving to advance the development of this technology.^509^ Specific commercial examples include providing medical records, assisting patients of different cultural levels in reading documents, providing access to “real-world evidence” to guide patient care, and thus guiding a new wave of clinical decision-support technologies.^509^

Patients can also interact with commercially available chatbots to guide their rehabilitation care. Several cases indicated that chatbots could effectively assist patients in understanding their diagnosis, treatment, monitoring plans, and subsequent healthcare plans during the interactive process.^510^ In addition, not all such applications are based on AI technology. For example, an interactive smartphone application can effectively help people with prostate cancer and breast cancer reduce the side effects caused by radiotherapy and chemotherapy.^511,512^

Most digital technologies survey users through mobile applications and provide advice on areas such as mental health, substance use, and sleep disorders. Other data that may need additional input from patients (such as blood sugar and blood pressure data) mainly refer to the guidance of some common complications, such as diabetes and hypertension,^513^ or even direct control. At present, the application of these digital technologies in oncology mainly focuses on mental health care and reducing adverse effects. In addition to many other digital healthcare services, this therapy can also be included in digital healthcare prescriptions.^514^

### Psychological and social support

The mental health of cancer patients plays an essential role in their recovery process. For breast cancer patients, since the disease is within the breast, in addition to becoming suppressed, anxious, and fearful, breast cancer patients are usually easily unconfident in their appearance. Therefore, it is essential to carry out appropriate psychological interventions for breast cancer patients. An early meta-analysis showed the importance of psychoeducational support on the quality of life and rehabilitation of breast cancer patients.^515^ Currently, there are many types of psychological intervention methods for breast cancer patients, which include supportive psychotherapy, cognitive behavior therapy, family therapy, etc.

Acceptance and Commitment Therapy (ACT) is a psychological therapy based on relational framework theory and is the latest development of cognitive-behavioral therapy. This therapy integrates Eastern culture and has good public applicability in China. ACT’s six core treatment processes include focusing on the present, dissociation, acceptance, self-reflection, feeling value, and commitment to action. Enhancing mental flexibility and quality of life can be achieved through actively accepting one’s emotions and physical sensations, minimizing avoidance behavior, and clarifying value-inspiring actions.^516,517^ A recent 3-arm pilot randomized controlled trial indicated that ACT could significantly reduce the fear of cancer recurrence rate at each time point compared with baseline.^518^ ACT also showed promise in improving sleep-related symptoms and fatigue in metastatic breast cancer patients.^519^

Group psychotherapy refers to several breast cancer patients forming a treatment group, and clinicians will provide the group with relevant knowledge about breast cancer rehabilitation and teach them the corresponding skills. Patients will obtain the opportunity to vent their negative emotions to each other and get mutual support within the group, thus increasing the confidence of rehabilitation among patients.^520,521^ Similar results were also found in metastatic breast cancer patients.^522^

In addition to the above psychological intervention, breast cancer is also a family issue.^523^ It should be noted that psychological therapy for patients and their partners (including counseling and guidance on sexual rehabilitation) is of great significance in improving their communication, maintaining good relationships, and promoting the psychological and physical recovery of patients. A recent randomized study has indicated the promise of family-centered positive psychological intervention in treating breast cancer patients.^524^

### Diet and lifestyle intervention

#### Exercise

Among the lifestyle interventions that breast cancer patients can engage in, exercise is the most important one that can effectively reduce recurrence and treatment-induced symptoms. Studies have proven that exercise could reduce breast cancer recurrence. Exercise has been proven to facilitate recovery after breast cancer surgery in a randomized controlled trial. The recovery of shoulder function has been achieved in 67.9% in the exercise group compared to 3.6% in the control group at one month postsurgery.^525^ Similar results were also found in an earlier study.^526^ A meta-analysis demonstrated that exercise during chemotherapy and/or radiotherapy could improve improved fatigue, depression, and quality of life in breast cancer patients.^527^ Another study has demonstrated that exercise led to improvement in aromatase inhibitors-induced arthralgia.^528^ It was also reported that exercise can change the tumor microenvironment of breast cancer,^529^ which indicated the potential of combining immunotherapy. Exercise on specific body part, such as upper limb exercises, can also help alleviate lymph node edema in breast cancer patients.^530^ A subtyping study demonstrated that HR+/HER2− breast cancer was more responsive to exercise than other subtypes,^531^ while another basic study has pointed out that exercise did not impact the proliferation of breast tumors but has changed their gene expression.^532^ Interestingly, it was reported that the benefit of exercise on breast cancer patients was partially mediated by changes in insulin levels.^533^

The American Society of Clinical Oncology also suggests aerobic exercise to reduce treatment-associated side effects.^534^ For patients who received chemotherapy, exercise could significantly improve cognition and fatigue, sleep quality, and quality of life.^535^ The 12-month exercise program significantly reduced pain scores by 30% when treated with aromatase inhibitors.^528^

#### Diet and nutritional supplements

Currently, research has not found any specific diet that can significantly improve the quality of life of cancer patients after treatment, but we can still make general recommendations. One study indicated that compared to the traditional standard diet, a low-fat diet with increased whole grains, vegetables, and fruits could significantly improve the OS of breast cancer patients.^536^ Other studies have found that high amounts of saturated and trans-fats were associated with increased all-cause mortality. Thus, a plant-enriched diet with whole grains and healthy fats is recommended. In the meantime, artificial sweeteners and processed meats should be avoided.^537^ A ketogenic diet induced a greater reduction in tumor size compared to the control group (27 versus 6 mm) in a randomized controlled study.^538^ Fruit and vegetables, as a well-known diet good for health, have also been observed to reduce breast cancer risk in a recent meta-analysis.^539^

Although guidelines did not recommend dietary supplements for preventing and/or treating cancer in case of no poor diet or nutrient deficiency,^540,541^ certain nutritional supplements can be applied for controlling symptoms. For example, treating vitamin D deficiency may help improve bone health and improve breast cancer outcomes,^542^ while for breast cancer patients who received chemotherapy, Wisconsin ginseng is shown to reduce fatigue.^543^ It should also be noted that certain supplements are harmful and should avoided. For example, iron and Vitamin B_12_ during chemotherapy can reduce breast cancer recurrence and death.^544^ Acetyl-L carnitine could worsen neuropathy induced by taxane.^545^ In some studies, supplemental antioxidants had been reported to worsen cancer outcomes and thus should also be avoided.^546^

## Conclusions and perspectives

Breast cancer remains a significant global health challenge characterized by complex etiology and diverse clinical presentations. This comprehensive review explores the epidemiology and risk factors of breast cancer, highlighting vulnerable populations and contributions from environmental and genetic factors. Pathophysiological and molecular subtypes of breast cancer are summarized, emphasizing disease heterogeneity and the importance of personalized therapies. Mechanisms driving breast cancer progression are discussed, showcasing recent research elucidating intricate pathways.

Advancements in diagnostic technologies, such as improved imaging and molecular diagnostics, significantly enhance early detection and accuracy, reducing misdiagnosis and missed cases. These advancements aid in optimizing treatment strategies and ensuring timely and appropriate care for patients. Treatment paradigms for breast cancer continue to evolve rapidly, with increasing focus on minimizing overtreatment and advancing precision and personalized medicine principles in clinical practice. Emerging therapies offer new hope for improving patient survival rates. Additionally, the importance of maintaining quality of life and long-term management for breast cancer patients is underscored, addressing challenges in managing treatment side effects and psychological impacts and preventing recurrence to enhance long-term survival rates.

Looking forward, several critical areas necessitate further research and exploration. These include enhancing public awareness to address the high incidence of breast cancer, understanding the molecular mechanisms underlying metastasis and recurrence, leveraging advancements in genomics, proteomics, and metabolomics to develop more personalized and effective treatments, and bridging the gap between basic research findings and clinical applications. Establishing interdisciplinary collaboration platforms is crucial to facilitate the seamless integration of scientific discoveries with clinical trials, driving innovation and progress in breast cancer treatment.

In summary, breast cancer, as one of the most prevalent malignancies among women, remains a major focus of extensive basic and clinical research aimed at unraveling its complexity. Future efforts should continue to prioritize these key research areas to achieve more effective advancements in the understanding, treatment, and prevention of breast cancer.
